# Human Dendritic Cell Subsets, Ontogeny, and Impact on HIV Infection

**DOI:** 10.3389/fimmu.2019.01088

**Published:** 2019-05-16

**Authors:** Jake William Rhodes, Orion Tong, Andrew Nicholas Harman, Stuart Grant Turville

**Affiliations:** ^1^Centre for Virus Research, The Westmead Institute for Medical Research, Sydney, NSW, Australia; ^2^Sydney Medical School, The University of Sydney, Sydney, NSW, Australia; ^3^Discipline of Applied Medical Sciences, School of Medical Sciences, The University of Sydney, Sydney, NSW, Australia; ^4^University of New South Wales, Sydney, NSW, Australia; ^5^Kirby Institute, Kensington, NSW, Australia

**Keywords:** HIV, dendritic cells, in trans, myeloid, plasmacytoid

## Abstract

Dendritic cells (DCs) play important roles in orchestrating host immunity against invading pathogens, representing one of the first responders to infection by mucosal invaders. From their discovery by Ralph Steinman in the 1970s followed shortly after with descriptions of their *in vivo* diversity and distribution by Derek Hart, we are still continuing to progressively elucidate the spectrum of DCs present in various anatomical compartments. With the power of high-dimensional approaches such as single-cell sequencing and multiparameter cytometry, recent studies have shed new light on the identities and functions of DC subtypes. Notable examples include the reclassification of plasmacytoid DCs as purely interferon-producing cells and re-evaluation of intestinal conventional DCs and macrophages as derived from monocyte precursors. Collectively, these observations have changed how we view these cells not only in steady-state immunity but also during disease and infection. In this review, we will discuss the current landscape of DCs and their ontogeny, and how this influences our understanding of their roles during HIV infection.

## Introduction

The first description of a dendritic cell originated from Paul Langerhans in 1868 based on his identification of skin-based cells (now known as Langerhans cells) that had a striking dendritic or “tree-like” morphology (1). Following the study and characterization of the mononuclear phagocyte system, seminal work by Ralph Steinman and Zanvil Cohn led to the identification of phagocytic cells in the spleen that could also induce antibody responses, which were then formally named “dendritic cells” (DCs) in 1973 (2). DCs were further shown to express high levels of surface major histocompatibility complex (MHC) molecules and potently induced proliferation of naïve T cells (3). In addition to the spleen, DCs were described across multiple peripheral tissue compartments by Hart and Fabre (4), and antigen-retaining cells also identified within B-cell follicles by Szakal and Hanna (5) and Nossal et al. (follicular DCs) (6). The DC family was subsequently expanded in 1994 to include a plasma-like cell (plasmacytoid DC) first described in 1958 (7) found to develop DC-like features upon culture by O'Doherty in the Steinman lab (8), and later identified in 1999 as the principal type I interferon-producing cell in blood by Cella et al. (9) and Siegal et al. (10). It was also discovered in the late 1980s that DCs (then described as veiled accessory cells) could be derived from cultured monocytes (11), meaning by the turn of the century the variety of DCs encompassed Langerhans cells and interstitial DCs in non-lymphoid tissue, Steinman-Cohn DCs, and follicular DCs in lymphoid tissue, Steinman-Cohn DCs, and pDCs in circulation and monocyte-derived DCs.

What defines a DC has been subject to extensive change over time but typically refers to their ability to take up antigens and present them to antigen-specific naïve T cells, leading to activation and proliferation of the T cell. As the complexity of DC subsets has grown, so too has our appreciation of their various functions outside of T cell priming and within different disease contexts, such as during infection with a viral pathogen such as human immunodeficiency virus (HIV). In this review, we attempt to summarize the current repertoire and ontogeny of DCs in peripheral blood and tissue of the anogenital tract (sites where the sexual transmission of HIV occurs) and provide an up-to-date look into their role in propagating and defending against HIV infection.

## Dendritic Cell Subsets and Ontogeny

The ability to define and describe key immune cells has rapidly improved in recent years due to advances in single-cell technologies, which allow us to accurately discern subtle transcriptional and functional differences in the highly diverse cells of the immune system. Although microscopy and flow cytometry approaches have provided a wealth of information about immunity during steady-state and disease, they are constrained by a limited number of assessible parameters and require prior knowledge about specific antigens of interest. The advent of increasingly high-parameter techniques, particularly single-cell RNA sequencing which captures the entire transcriptome (17,000+ genes) of a single cell at a precise moment in time, has enabled rigorous and unbiased classification of immune cells (12–20) and their developmental processes (21–24). A large number of studies leveraging the power of these high-dimensional single-cell technologies have focused on the landscape of DCs given their rarity and reported heterogeneity across peripheral blood and tissue.

### Dendritic Cells in Peripheral Blood

**Figure 1:**
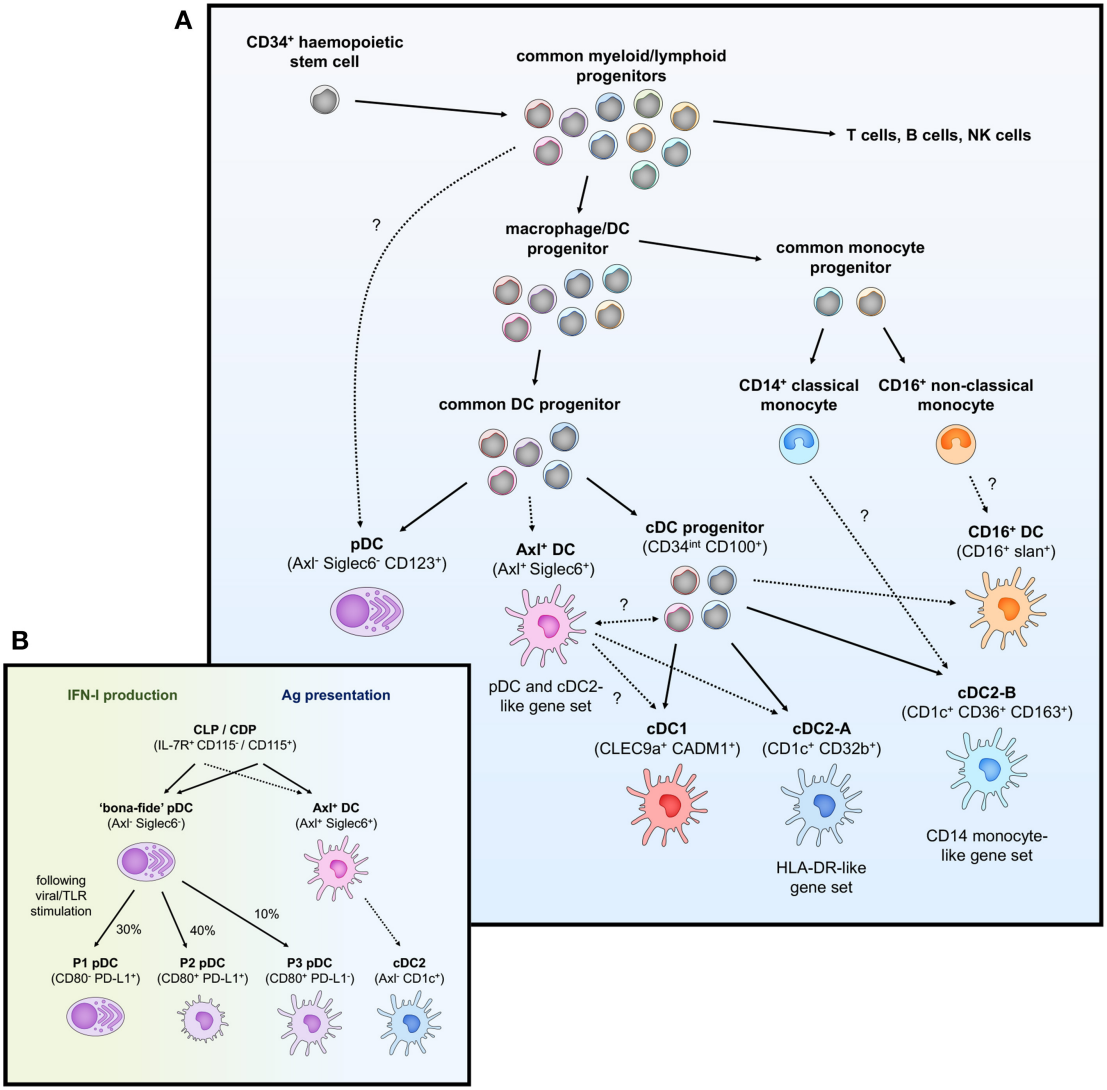
Overview of peripheral blood DC subsets and ontogeny. **(A)** Highly proliferative and self-renewing CD34^+^ hematopoietic stem cells produce early progenitors each primed toward distinct cell fates. These progenitors pass through several shared phenotypes to create heterogenous populations of macrophage/DC progenitors, common DC progenitors, cDC progenitors to eventually generate functional DCs (pDCs, Axl^+^ DCs, cDC1, cDC2-A, cDC2-B, and CD16^+^ DC). Whether these DCs represent fully-differentiated cells is contentious—Axl^+^ DCs have been separately described to be a fully functional end-stage cell, be capable of acquiring a cDC2-like phenotype, or represent a more mature cDC progenitor that can further differentiate into cDC1 and cDC2. Their relationship to a primitive cDC progenitor (CD34^int^ CD100^+^) is also not clear. The relationship between cDC2-B and CD16^+^ DC with monocytes is also ambiguous at the present time. **(B)** pDCs (CD123^+^ BDCA2^+^) have previously been ascribed both IFN-I production and antigen (Ag) presentation properties. The identification of Axl^+^ DCs which also express typical pDC markers has clarified these functions, which can now be separately attributed to Axl^−^ pDCs and Axl^+^ DCs, respectively. However, Axl^−^ pDCs can also differentiate into Ag-presenting pDCs following stimulation (P2 and P3 pDCs) that have limited capacity for IFN-I production, creating some complication for accurately demarcating cells with IFN-I ability and T cell stimulation in the CD123^+^ population.

Traditionally, the DC population in peripheral blood has been classified into two lineages based on phenotypic and functional characteristics. Conventional DCs (cDCs), also known as myeloid DCs, can be defined as CD11c^+^ CD123^−^ and are specialized at antigen uptake and presentation to naïve T cells, thus representing the “typical” antigen-presenting DC that primes adaptive immunity. cDCs have previously been subdivided into two subsets (cDC1 and cDC2) based on homology to murine equivalents (CD8α^+^/CD103^+^ and CD11b^+^ DCs respectively) (25) and the differential expression of key transcription factors that drive their development; interferon regulatory factor (IRF)8, basic leucine zipper transcriptional factor ATF-like 3 (BATF3) and DNA-binding protein inhibitor 2 (ID2) for cDC1 and IRF4, Neurogenic locus notch homolog protein 2 (Notch2) and Kruppel-like factor 4 (KLF4) for cDC2 (26, 27). In contrast, plasmacytoid DCs (pDCs) are CD11c^−^ CD123^+^ cells best characterized for their type I interferon (IFN-I) production during viral infection but can also perform a variety of other functions including T cell stimulation and pro-inflammatory cytokine and chemokine secretion (28). Other populations of peripheral blood DC (either distinct from or further subsets of cDCs and pDCs) have also been described based on the expression of various other markers including CD2, CD5, CD16, CD34, and Slan (29–35) but have not been confirmed as distinct subsets by detailed transcriptomic or lineage analyses to date. By extensively profiling the DC population through unbiased single-cell RNA sequencing and flow/mass cytometry, several key studies have identified 6 distinct populations of blood DCs, spanning four putative cDC subsets, one subset of pDCs, and one intermediary subset that spans both cDC and pDC-like gene expression (Figure 1A and Table 1) (14, 17, 36).

**Table 1:** Human blood and tissue dendritic cell phenotypes.

**Blood**				**Tissue**			
**Population**	**Subsets**	**Markers**		**Population**	**Subsets**	**Markers**	
cDC1 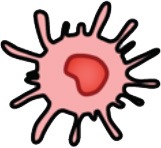 (IRF8, BATF3, ID2)	cDC1	HLA-DR^+^	**Clec9a**^**+**^	cDC1 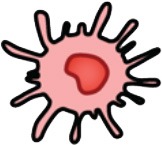	Dermal cDC1	HLA-DR^+^	CADM1^+^
		CD11c^lo^	**CD141**^**+**^			**CD141**^**++**^	Clec9a^+^
		CD33^+^	**XCR1**^**+**^			**CD1c**^**−/low**^	XCR1^+^
			**CADM1**^**+**^			**CD11c**^**low**^	CD103^+^ (intestinal)
						**CD14**^**−**^	SIRPα^−^
						Langerin^−^	
	
cDC2-A 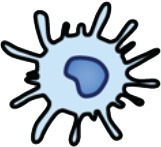 (IRF4, Notch2, KLF4)	cDC2-A	HLA-DR^++^	**CD1c**^**++**^	cDC2 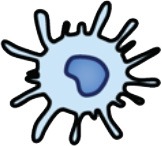	Dermal Langerin^−^ cDC2	HLA-DR^+^	**Langerin**^**−**^
		CD11c^++^	**CD32b**^**+**^			**CD1a**^**+**^	DC-SIGN^−^
		CD33^+^	CD5^hi^			**CD11c**^**+**^	CD11b^+^
		CD11b^+^ SIRPα^+^	HLA-DR-like gene set			**CD141**^**−**^ SIRPα+	CD1c^+^ CD103^+^ (intestinal)
	cDC2-B	HLA-DR^+^	**CD1c**^**+**^		Dermal Langerin^+^ cDC2	HLA-DR^+^	**Langerin**^**+**^
		CD11c^+^	**CD36**^**+**^			**CD1a**^**+**^	DC-SIGN^−^
		CD33^+^	**CD163**^**+**^			**CD11c**^**+**^	CD11b^+^
		CD11b^+^ CD5^lo^	CD14-mono-like gene set			**CD141**^**−**^ SIRPα+	CD1c^+^ CD103^+^ (intestinal)
	
CD16^+^ DC 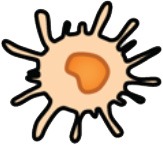	CD16^+^ DC	HLA-DR^+^	**CD16**^**+**^	CD14^+^ cells 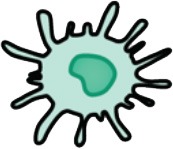	CD14^+^ MDM	HLA-DR^+^	**Autofluorescence**^**−**^
		CD11c^+^	**Slan**^**+**^			**CD1c**^**−/low**^	CD11c^+^
		CD141^−^	CD86^hi^			**CD14**^**+**^	CD11b^−^
		CD1c^−^ CD33^int^	CD16-mono-like gene set			CD16^−^	DC-SIGN^+^
					CD14^+^ CD1c^+^	HLA-DR^+^	**Autofluorescence**^**−**^
						**CD1c**^**+**^	CD11c^+^
						**CD14**^**+**^	CD11b^−^
						CD16^−^	DC-SIGN^+^
					CD14^+^ macrophages	HLA-DR^+^	**Autofluorescence**^**+**^
						**FXIIIA**^**+**^	CD64^+^
						**CD14**^**+**^	DC-SIGN^+^
Axl^+^ DC 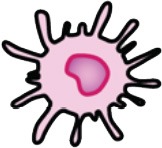 (ID2, TCF4)	CD123^+^ Axl^+^ DC	HLA-DR^+^ CD11c^int^ CD1c^−^ CD123^+^ BDCA-2^+^ BDCA-4^int^ CD2^hi^ CD5^+^	**Axl**^**++**^ **Siglec6**^**++**^ **Siglec1**^**+**^ **Siglec2**^**+**^ CD45RA^+^ CD33^int^ pDC-like gene set	LCs 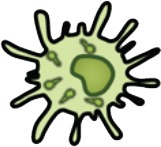	LCs	HLA-DR^+^ **CD1a**^**++**^ **Langerin**^**+**^ CD11c^lo^ CD1c^+^	**Birbeck granules**^**+**^ E-Cadherin^+^ DC-SIGN^−^ EpCAM^+^
				CD1a^+^ VEDCs 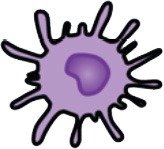	CD1a^+^ VEDCs	HLA-DR^+^	**Birbeck granules**^**−**^
						**CD1a**^**+**^	DC-SIGN^−^
	CD11c^+^ Axl^+^ DC	HLA-DR^++^ CD11c^+^ CD1c^int/+^ CD123^int^ BDCA-2^int^ BDCA-4^lo^ CD2^hi^ CD5^+^	**Axl**^**+**^ **Siglec6**^**+**^ **Siglec1**^**int**^ **Siglec2**^**+**^ CD45RA^int^ CD33^+^ cDC2-like gene set			**Langerin**^**+**^ **CD11c**^**+**^	CD14^−^
				IDECs 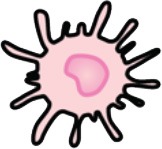	IDECs	HLA-DR^+^ **CD1a**^**+/lo**^ **CD11c**^**+**^ FCeR1^+^	**Birbeck granules**^**−**^ CD36^+^ CD32^+/lo^ CD11b^+/lo^
pDC 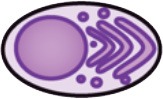 (TCF4, IRF7, IRF8)	pDC	HLA-DR^lo^	**CD123**^**hi**^	Intestinal Macrophages 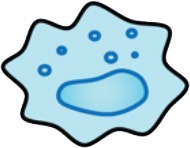	MF1	**HLA-DR**^**+**^	CD11b^+^
		CD11c^−^	**BDCA-2**^**hi**^			**CD14**^**+**^	CD206^−^
		CD33^−^	**BDCA-4**^**+**^			**CD11c**^**+**^	CD1c^+/lo^
		CD2^lo/hi^	Axl^−^		MF2	**HLA-DR**^**++**^	CD11b^+^
		CD5^−^	Siglec6^−^			**CD14**^**+**^	CD206^+^
		CD45RA^+^	CCR7^+^			**CD11c**^**+**^	CD1c^+^
					MF3	HLA-DR^+^	**CD11b**^**−**^
						**CD14**^**+**^	CD206^+^
						**CD11c**^**−**^	CD1c^−^
					MF4	HLA-DR^+^	**CD11b**^**+**^
						**CD14**^**+**^	CD206^+^
						**CD11c**^**−**^	CD1c^−^

*Known transcription factors in brackets. Key distinguishing markers highlighted in bold*.

#### Conventional DC1

Peripheral blood cDC1s are a single and discrete population of DC and can be best identified by expression of C-type lectin-like receptor (Clec)9A and cell adhesion molecule 1 (CADM1) (found almost exclusively on cDC1) (14, 36), and also express high levels of CD141 and XCR1, which is how they have commonly been identified (37, 38). cDC1s represent a rare population of DC [~0.05% of peripheral blood mononuclear cells (PBMCs)] (39) and are mainly noted for their superior cross-presentation ability compared to other DC subsets (37, 39), efficiently priming CD8+ T cells against extracellular antigens such as bacterial and viral pathogens that DCs cannot become infected by. In addition, cDC1s can efficiently present necrotic antigens to T cells (39), in part mediated by the binding of Clec9A to extracellular actin exposed during the process of cellular necrosis (40, 41). cDC1s also express high levels of Toll-like receptor (TLR)3, TLR9, and TLR10 (14, 42, 43) which allows them to detect intracellular dsRNA and DNA, and leads to IRF3-dependent production of type I IFNs and interleukin (IL)-12 (37, 44).

#### Conventional DC2

In contrast to a single population of cDC1s, peripheral blood cDC2s can be further subdivided into two subsets: cDC2-A and cDC2-B [referred to as DC2 and DC3, respectively by Villani et al. (14)]. Although both cDC2 subsets can be characterized by CD1c expression, cDC2-A (CD32b^+^) are distinguished by higher levels of CD11c, CD1c, and MHC class II genes, whilst cDC2-B (CD36^+^ CD163^+^) have increased expression of inflammatory genes and a similar expression signature to that of CD14^+^ classical monocytes (14). These two subsets appear to match previous reports of cDC2s divided into CD5^hi/lo^ populations (31) (corresponding to cDC2-A/B, respectively) as well as putative CD14^+^ and CD163^+^ subsets of CD1c-expressing cells (cDC2-B) (45, 46). Furthermore, from their mass-cytometry-based examination of human blood DCs, Hernandez et al. also describe several populations of cDC2s separated by CD163 and signal regulatory protein α (SIRPα) expression (cluster 3–5) (17), with cluster 3 (CD11c^++^ CD163^−^) and cluster 5 (CD11c^+^ CD163^+^) loosely corresponding to cDC2-A/B. Interestingly, cluster 4, as defined by intermediate CD163 expression and high levels of CD11c (like cDC2-A) but low CD1c (like cDC2-B), may represent innate plasticity between cDC2-A/B, which is unsurprising given the high interindividual variation in the circulating cDC2 population (17).

cDC2s represent the major subset of myeloid DC in blood and act as potent stimulators of naïve T cells. They also express a wide range of lectins such as Clec4A, Clec10A, Clec12A, and DEC205 (Clec13B) (47–50). They also express TLR2, 4–6, 8 & 9 and correspondingly produce a wide range of soluble factors in response to TLR stimulation such as tumor necrosis factor (TNF)- α, IL-1, IL-6, IL-8, IL-12, and IL-18, and chemokines such as CCL3, CCL4, and CXCL8 (14, 51). Consistent with their DC-like gene signature, CD5^hi^ cDC2-A appear to undergo increased CCR7-dependent migration, stimulate higher naïve T cell proliferation and produce higher levels of innate cytokines than cDC2-B (14, 31). cDC2-A/B also appear to induce different T helper (Th) cell polarization [Th2, Th17, Th22, and regulatory T cell (Treg) vs. Th1, respectively] (31), thus directing the immune system in quite distinct directions.

#### CD16^+^ DC

A fourth subset of CD11c-expressing DC has also been identified which lacks expression of either CD141 or CD1c and can instead be identified by CD16 expression [referred to as DC4 by Villani et al. (14)]. These DCs may be similar to the CD16^+^ DCs described earlier by MacDonald et al. (34) which have high CD86 and CD40 expression but lower levels of HLA-DR and possess potent T cell stimulatory capacity. CD16^+^ DCs have previously been reported to produce large amounts of inflammatory cytokines in response to TLR agonists such as TNF-α, IL-6, and IL-12 (51–53), and so appear to occupy a pro-inflammatory role. However, it is important to note that CD16^+^ CD11c^+^ cells may not represent actual DCs but rather a subset of CD16^+^ non-classical monocyte. DC4s identified by Villani et al., (14) have marked differences in IFN-I signaling/viral response and leukocyte migration gene expression to CD16^+^ non-classical monocytes despite other similarities in transcriptional profile. Based on surface marker expression however, such as comparative levels of CD16, CD11b, CD14, and CD36, DC4s may also describe Slan^+^ cells (previously thought to be a CD16-expressing DC, but now associated with monocyte identity) (54–56). Recent work examining DC4 phenotype and T cell stimulatory function also suggests they represent a subset of Slan-expressing (73%) CD14^dim/−^ CD16^++^ monocyte (57). Regardless, further studies are needed to understand the phenotypic and functional nuances of this cell subset, particularly due to the exclusion of CD14 and CD16-expressing cells in most other recent high-dimensional studies of the DC repertoire (17, 36).

#### Plasmacytoid DC

Traditionally defined as CD123^+^ BDCA-2^+^ cells, pDCs have also been redefined by single-cell RNA sequencing as encompassing two DC subsets. “Bona-fide” pDCs are presently defined as a single population of IFN-I-producing cells lacking the capacity to stimulate T cell responses whilst in an immature state and are distinct from a small proportion of “contaminating” CD123^+^ myeloid DCs (referred to as Axl^+^ DCs hereafter) which are unable to produce IFN-I but can potently activate T cells (14, 17, 36). Based on phenotype, Axl^+^ DCs match previous descriptions of a CD2^hi^ CD5^+^ CD81^+^ “subset” of pDC that produces little to no IFN-I but induces higher T cell stimulation than CD2^lo^ counterparts (14, 36). Although pDCs express slightly higher levels of typical pDC markers (CD123, BDCA-2, and particularly CD304/BDCA-4) than Axl^+^ DCs (14, 36), this cannot be reliably used to separate the two populations. Instead, pDCs can be gated as negative for specific Axl^+^ DC markers (Axl, Siglec6) as well as myeloid markers (CD11c, CD33, CX3CR1) expressed by the Axl^+^ DCs. pDCs can also be easily identified by their secretory morphology with a round shape, eccentric nuclei, pronounced endoplasmic reticulum, and pale Golgi zone (14, 36).

pDCs have previously been attributed a variety of functions ranging from the production of antiviral type I and III IFN, priming NK cell activation via IL-12 and IL-18 secretion, and antigen presentation and priming of T cells [reviewed by Swiecki and Colonna (28)]. With the discovery of contaminating myeloid DCs within the CD123^+^ gate used to previously define pDCs, many of these reported cDC-like properties such as T and B cell activation potential and IL-12 production can no longer truly be attributed to pDCs. “Bona-fide” pDCs still represent key drivers behind type I and III IFN responses particularly to viral pathogens through their endosomal expression of TLR7 and TLR9 (which sense ssRNA and dsDNA, respectively) and high constitutive expression of IRF7 (14, 58). Activation of TLR7/9 also induces NF-κB expression, leading to the production of TNF-α and IL-6 by pDCs, reviewed in Gilliet et al. (59). Other soluble factors produced by “bona-fide” pDCs upon TLR stimulation include the chemokines CCL3, CCL4, CCL5, IL-8, CXCL10, and CXCL11 (14, 36). In contrast to the reported inability of immature pDCs to stimulate T cells, several recent studies have described allogeneic T cell proliferation potential by Axl^−^ “bona-fide” pDCs activated with CD40L and IL-3, influenza virus or CpG oligonucleotides (17, 60), suggesting activated pDCs may be able to differentiate into cDC-like cells. Indeed, Alculumbre et al. identified three stable subpopulations of activated Axl^−^ pDCs defined by PD-L1 and CD80 expression; PD-L1^+^ CD80^−^ cells retained a plasmacytoid morphology and high IFN-I production whilst PD-L1^−^ CD80^+^ cells developed a dendritic morphology, had increased CCR7 expression and were capable of T cell activation and Th2 polarization but were unable to produce IFN-I (Figure 1B) (60). PD-L1^+^ CD80^+^ cells represented an intermediate state between typical pDC and cDC-like functions. Altogether, this suggests that even the Axl^−^ “bona-fide” pDC compartment has a large degree of heterogeneity and is consistent with previous reports that only a small fraction of pDCs produce IFN-I in response to stimuli (61, 62).

#### Axl^+^ DC

The newly described CD123^+^ myeloid DCs can be defined by the expression of unique discriminatory markers (Axl, Siglec6) as well as a blend of typical pDC (CD123, BDCA-2, BDCA-4, and CD45RA) and cDC2 markers (CD11c, CD33, CX3CR1, CD1c, CD2). They have been alternatively identified as Axl^+^ Siglec6^+^ AS DCs by Villani et al. (14), CD33^+^ CD45RA^+^ CD123^+^ pre-DCs by Zoccali et al. (36) and Axl^+^ cells expressing either CD11c, CD123 or CD1c (or a combination thereof) by Alcantara-Hernandez et al. (17). Clustering analysis indicates that they span a continuum of pDC-like and cDC2-like states which can be identified through CD123/CD11c expression: CD123^hi^ CD11c^lo^ cells have a transcriptomic profile more similar to pDCs, and CD123^lo^ CD11c^hi^ cells appear more closely related to cDC2s (14). This is consistent with variation across the Axl^+^ DC population in TCF4 and ID2 expression, key transcription factors responsible for maintaining pDC and cDC commitment, respectively. Despite their phenotypic similarity with pDCs, Axl^+^ DCs cells are unable to produce IFN-I and resemble cDC2s in terms of basic function and morphology. Consistent with their potent allostimulatory potential, they express high levels of CD86 and HLA-DR, but also express a unique pattern of glycan-binding lectins (Siglec1, Siglec2, and Siglec6) and Axl, which binds apoptotic cells, indicating they have distinct functions outside of antigen presentation and T cell stimulation (14, 33). Like cDC2s, Axl^+^ DCs express TLR4 and TLR5, as well as IRF4 and IRF8, indicative of the capacity to respond to bacterial pathogens with cytokine and chemokine production. However, they have limited expression of TLR7, IRF7 and other genes expressed by pDCs associated with IFN-I production and secretion (*DERL3, LAMP5*, and *SCAMP5*), which corresponds with their inability to produce IFN-I in response to TLR7/9 stimulation (14, 36). Given their recent discovery, the full spectrum of their immune functions remains to be elucidated.

### Dendritic Cell Ontogeny

Under the traditional model of haematopoiesis via progressive fate “decisions,” both cDCs and pDCs develop from CD34^+^ haematopoietic stem cells (HSC) which transition into the common myeloid progenitor (CMP), excluding lymphoid lineage potential, which then differentiates into the macrophage-DC progenitor (MDP) and excludes granulocyte potential (63–68). The MDP further differentiates into the common DC progenitor (CDP), thus excluding monocyte and macrophage lineages, with the expression of zinc finger and BTB domain containing 46 (ZBTB46) and ID2 driving specification into a cDC-precursor whilst transcription factor 4 (TCF4) expression leads to pDC commitment (69–72). Further differentiation of the cDC-precursor into cDC1s and cDC2s is dependent on the expression of key transcription factors associated with each subset (BATF3 and IRF8 for cDC1 or IRF4 and KLF4 for cDC2 as previously mentioned) (73–76).

With the identification of several new DC subsets (cDC2-A/B, CD16^+^ DCs, and Axl^+^ DCs), the process that might generate these cells and its relation to existing notions of DC development is unclear (Figure 1A). Given the transcriptional relationship between cDC2-B and CD16^+^ DCs with CD14^+^/CD16^+^ monocytes, respectively, it is tempting to speculate they may be derived from a monocytic origin. DC-like cells can be generated by differentiation of monocytes into monocyte-derived DCs (MDDCs), which can occur *in vitro* via IL-4 and granulocyte-macrophage colony stimulatory factor (GM-CSF) supplementation or *in vivo* at tissue sites during inflammation (77–79), but whether MDDCs form in circulation during homeostasis is unclear. CD16^+^ MDDCs generated *in vitro* express several key genes associated with the DC4s described by Villani et al. (14), namely *SERPINA1, CD97, ITGAL*, and *TCF7L2* (80), but CD14^+^ MDDCs appear to transcriptionally align with CD14^+^ DCs in skin rather than CD14^+^ blood monocytes (50). Further fate mapping and lineage tracing studies adopting the exact gating strategy used to describe these subsets would be valuable for confirming their exact ontogeny.

The origin and relationship of Axl^+^ DCs to other DCs remains controversial, particularly as to whether they represent a fully differentiated and functional DC or whether they exist as precursor cells to cDC1/2. Villani et al. identified that AS DCs in their study had a limited capacity for further proliferation, and functionally and morphologically resembled fully differentiated cDC2s (14). In addition, AS DCs were found to transition toward a cDC2 but not cDC1 phenotype over culture, indicating they do not represent a general cDC precursor. The distribution of Axl^+^ DCs also does not appear to correspond with previously identified cDC precursors, given Axl^+^ DCs cannot be identified in skin but are present in secondary lymphoid organs (17). In contrast, Zoccali et al. demonstrate that CD33^+^ CD45RA^+^ CD123^+^ cells (corresponding to Axl^+^ DCs), are cDC precursors (preDCs) and can differentiate into functional cDC1 and cDC2, and further identified committed pre-cDC1 (CADM1^+^) and pre-cDC2 (CD1c^+^) subsets of preDC (36). All of the preDC populations were capable of IL-12 and TNF-α production in response to TLR stimulation and induced robust T cell proliferation, reflecting that a precursor status does not preclude effector DC function. Interestingly, Axl^+^ DCs were examined in the CD141^−^ gate by Villani et al. and so it may be that pre-cDC1s were not captured in their analysis of AS DC differentiation potential leading to the observation that these cells could not transition into a cDC1 phenotype. As suggested by Bassler et al. (81), these uncertainties in Axl^+^ DC development and differentiation potential could be resolved by further examination of (1) whether AS DCs and preDCs completely overlap, and then using a unified sorting strategy for (2) differentiation assays and (3) comparative transcriptome and lineage mapping analysis. Finally, Villani et al. identified a CD34^int^ CD100^+^ circulating cDC progenitor, which appears morphologically primitive and lacks the ability to respond to FMS-like tyrosine kinase 3 ligand (Flt3L) or GM-CSF (both required for pre-cDC development) but is capable of producing both cDC1 and cDC2 (14). The potential relationship between this cDC progenitor and CD34^+^ haematopoietic stem cells remains intriguing, as is the observation that these cDC progenitors do not upregulate Axl or Siglec6 gene expression at any time over culture and differentiation, thus further complicating our understanding of the cellular origins of Axl^+^ DCs and their role in DC ontogeny.

Furthermore, recent studies have cast uncertainty over the myeloid progenitor identity of DCs, particularly pDCs given their morphological similarity to plasma B cells. pDCs have traditionally still been associated with a myeloid lineage, with evidence to show pDC commitment within common DC progenitors (82–85). However, the generation of pDCs from CDPs appears to be insufficient to account for the frequency of pDCs compared to cDCs *in vivo*, suggesting there may be other developmental pathways for pDCs. Several recent studies have described the generation of pDCs from a lymphoid lineage—Rodrigues et al. (24) demonstrate that the majority of IFN-I-producing pDCs (70–90% of immature splenic pDCs) are derived from a pDC-committed lymphoid progenitor (IL-7R^+^ CD115^−^) in mice that produces substantially more pDCs than CDPs (IL-7R^−^ CD115^+^). Furthermore, pDCs (Bst2^+^ CD45RA^+^) generated from these CDPs could only weakly produce IFN-I and were instead efficient antigen-presenting cells, and also expressed a combination of pDC and cDC-associated genes, leading to their description by Rodrigues et al. (24) as pDC-like cells rather than real pDCs. Indeed, these cells appear more reminiscent of Axl^+^ DCs and/or P3 CD80^+^ PD-L1^−^ pDCs. The pDC-biased lymphoid progenitors were also shown to contain a Ly6D^+^ SiglecH^−^ subset capable of generating both pDCs and B cells (24), consistent with a recent study using human adult bone marrow and single-cell RNA sequencing to demonstrate pDCs share a common progenitor population with B cells (23), further confirming a possible lymphoid lineage for pDCs. In contrast to these findings, recent single-cell tracking studies have reinforced a myeloid developmental pathway for pDCs; lineage tracing of CD115^+^ CDPs shows that most pDCs develop from this myeloid progenitor (86), and pDCs show similar development kinetics to other myeloid cells whereas pDC-biased progenitors arise before lymphoid counterparts (87, 88). Overall, increasing evidence suggests that pDCs can have a dual myelo-lymphoid origin which may dictate their functions when fully differentiated.

Studies across both humans and mice have increasingly shown that cDCs and putative Axl^+^ DCs can also be derived from both myeloid and lymphoid progenitors (89–91). This collectively reflects a paradigm shift in our understanding of haematopoiesis, moving away from a model of homogenous multipotent progenitors that bifurcate into distinct but rigid cell fates (i.e., MDP bifurcation into macrophage or dendritic cell fate) and instead toward one where lineage is imprinted early during development in early progenitors, possibly prior to the emergence of distinctive phenotypes (92–99). As such, progenitors tend to follow predetermined differentiation pathways and most demonstrate uni-lineage potential, with some bi- and multi-lineage progenitors capable of multiple fates (97). Thus, stages of DC development, such as a “common DC progenitor” likely represent a mixture of progenitors following pre-determined pDC or cDC1/2 commitment (Figure 1A), rather than a homogenous population that subsequently undergoes a fate decision. In summary, the complexity and subtlety in early haematopoiesis makes it difficult to accurately trace or predict DC development, and understanding the transcriptional programming that is required or sufficient to imprint DC subset-specific fates will be crucial to a complete appreciation of DC ontogeny.

### Dendritic Cells in Tissue

#### The Human Anogenital Tracts

**Figure 2:**
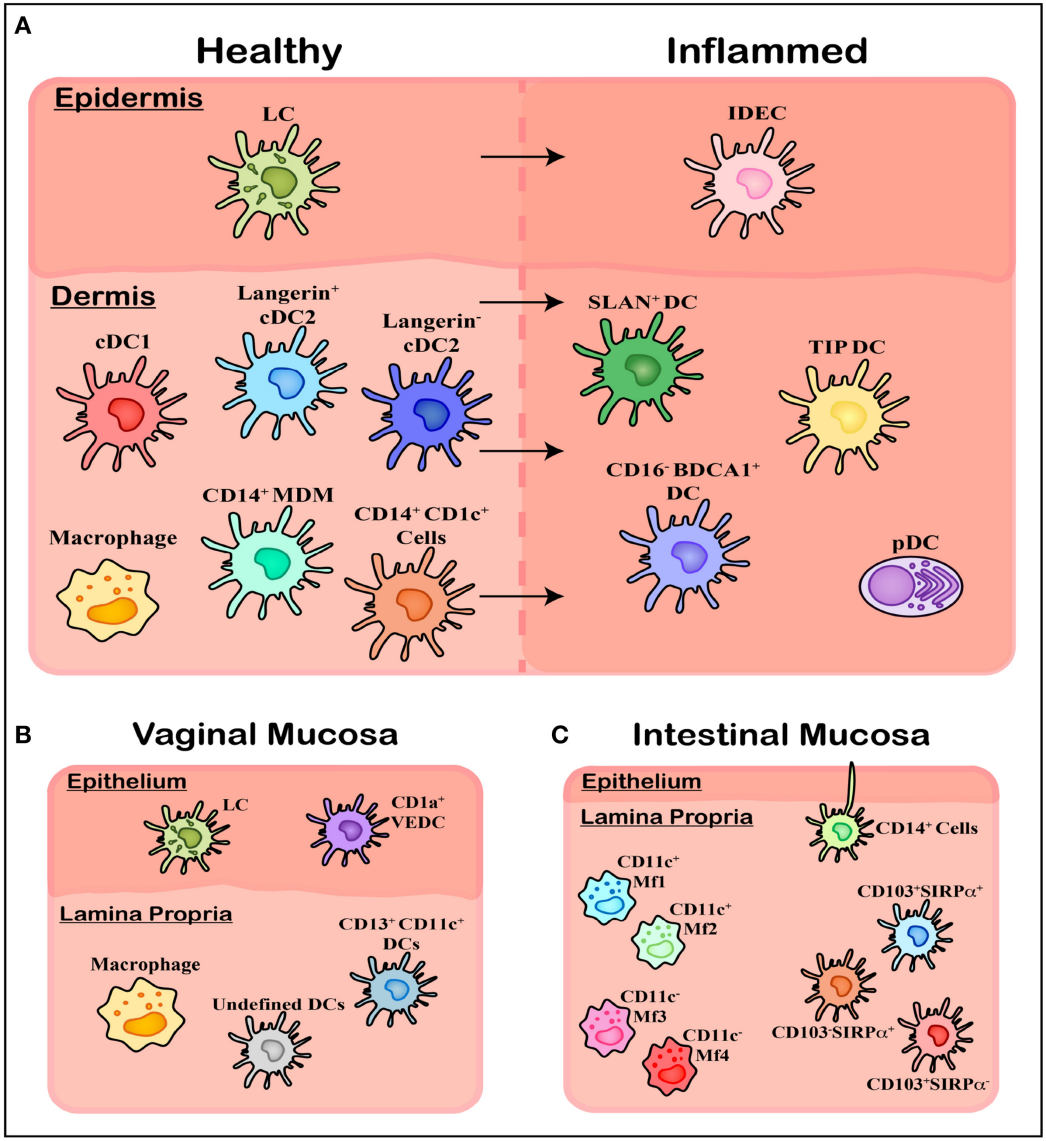
Overview of mononuclear phagocyte subsets in human tissue. **(A)** Within healthy skin, it is believed Langerhans Cells (LCs) are the solitary population, however the underlying connective tissue of the dermis contains a range of subsets including cDC1, cDC2 (langerin expressing and langerin negative populations), and CD14 expressing cells including tissue resident Macrophages, Monocyte-Derived Macrophages (MDMs), and an uncharacterized CD1c^+^ population. Upon inflammation these cells are also present however a number of other subsets of MNPs migrate in, including Inflammatory Dendritic Epidermal Cells (IDECs) in the epidermis, as well as pDCs, SLAN^+^ DCs, TIP DCs, and CD16^−^ BDCA1^+^ cells in the dermis. **(B)** Within the vagina another novel population has been identified in epithelial layer, termed CD1a^+^ VEDCs, while the underlying *lamina propria* has not been thoroughly characterized, with both macrophages and DCs present however exact subsets have not been extensively defined. **(C)** Within the *lamina propria* of intestinal tissue four separate CD14^+^ Macrophage populations (MF1-4) have been characterized by their CD11c^+^ expression, while undefined CD14^+^ cells have been shown to extend dendrites through the epithelium to sample luminal microbes. Finally, CD103 and SIRPα can define three population of DCs which align with blood cDC1s, cDC2s, and CD14^+^ monocytes.

The human genital and anorectal tracts (hereafter collectively referred to as the anogenital tract) are made up of three distinct tissue types that sexually transmitted pathogens such as HIV may encounter upon sexual transmission; skin, type I mucosa, and type II mucosa. These physical barriers differ in their physical, chemical, and biological makeup and therefore their permeability to infection. The different subsets of mononuclear phagocytes (MNPs) within these tissues are still being characterized but will be reviewed below and are summarized in Figure 2 and Table 1.

The skin is made up of two distinct layers, the outer epidermis and the underlying connective tissue layer called dermis, and covers the outer foreskin, glans penis, labia major & labia minora and the anal verge. It is made up of a thick stratified squamous epithelium with an outer layer of cornified cells, making it a formidable barrier to HIV infection. The vagina, ectocervix, inner foreskin, anal canal, and penile fossa navicularis are all covered by a type II mucosa, which similar to the skin, contains an outer epidermal layer with an underlying connective tissue layer called *lamina propria*. Unlike the skin, the type II mucosa lacks an outer layer of cornification and therefore has been shown to be more susceptible to HIV infection. The type I mucosa is considered the most susceptible tissue type of the human anogenital tract to HIV infection. Covering the endocervix, urethra, rectum, and colon, it is made up of a single layer of columnar epithelium overlaying the *lamina propria*.

### Method of Extraction of Dendritic Cells From Human Tissue

It is extremely important to note that the method of extraction of immune cells from tissue can have significant effects on the isolated cells functional state and also surface expression marker profile and therefore classification, causing potential conflicts in the literature. A notable example of this has been the correct identification of human tissue cDC1 (100–103). Botting et al. recently thoroughly tested a range of extraction techniques including a number of tissue dissociation enzymatic digestions and migration assays specifically looking at mononuclear phagocytes (104). They were able to show that a number of key surface identification markers and HIV entry receptors were enzymatically cleaved (e.g., CD11c, CD1c, CD14, CD4, CCR5), up-regulated (e.g., CD80, CD83, CD141) or down-regulated [e.g., Clec9A, mannose receptor (MR), dendritic cell-specific intercellular adhesion molecule-3-grabbing non-integrin (DC-SIGN)] depending on the method of extraction. This highlights the importance of taking note of the isolation methodology when investigating tissue mononuclear phagocytes and may help explain the ever-changing classification of these cells and conflicting results in the literature.

### Epidermal Dendritic Cells

The dendritic cells of the epidermis are one of the first cells to encounter incoming pathogens such as HIV. Historically it was believed that only a single population of human dendritic cells resided in this outer layer of tissue, however recent research has suggested this may not be the case.

#### Langerhans Cells

Since their discovery in 1868 the exact classification of Langerhans cells (LCs) has been controversial. While they are no longer considered to be a nerve cell as was first thought, it is still heavily debated whether LCs should be classified as DCs or macrophages. Functionally speaking LCs act as DCs, as they pick up foreign antigens, migrate out of tissue to the lymph node and present this antigen to naïve T cells, a process that macrophages do not undertake. However, ontogenically speaking LCs are more macrophage like, both sharing a common precursor with embryonic origins as well as having self-renewing abilities (105–107). Furthermore, while early *in vitro* studies reported the potential of monocytes to develop into LCs under GM-CSF and transforming growth factor beta (TGF-β) stimulation (108), it has more recently been shown that blood cDC2s have the potential to develop an LC-like phenotype under GM-CSF or thymic stromal lymphopoietin (TSLP) and TGF-β or bone morphogenetic protein 7 (BMP7) stimulation (109, 110).

LCs are characterized by their high expression of CD1a and Langerin as well as the presence of distinct Birbeck granules in their cytoplasm helping distinguish them from Langerin expressing dermal cDC2 (111) (Figure 2A). Being one of the first cells of the immune system coming into contact with invading pathogens, LCs are efficient at identifying foreign antigens, picking them up and presenting them to CD4 T-cells. This process is coordinated by the pattern recognition receptors (PRRs) they express including Langerin and TLRs 1, 2, 3, 5, 6, and 10 (112). Activated LCs have been shown to extend dendrites through keratinocyte tight junctions to sample and process antigens, while still maintaining barrier integrity by forming tight junctions themselves with the surrounding keratinocytes (113, 114). They have the ability to stimulate Th1, Th2, Th,17, and Th22 responses (115–118) making them highly immunogenic and adaptable to the wide range of antigens they encounter, while also showing immunosuppressive abilities under particular inflammatory conditions (119, 120).

#### CD1a^+^ Vaginal Epidermal Dendritic Cells (VEDCs)

More recently, while investigating HIV and its interactions with vaginal epidermal dendritic cells (VEDCs) Pena Cruz et al. identified a DC subset within healthy human epidermis of the vagina distinct from LCs (121) (Figure 2B). Like LCs, these cells were characterized as CD1a^+^ Langerin^+^ DC-SIGN^−^ but lacked Birbeck granules, thus distinguishing them from LCs. Given Langerin drives Birbeck granule formation (122, 123), the presence of Langerin without the latter feature is curious and supports other mechanisms for the formation of these LC-specific structures in addition to Langerin alone. Furthermore, these VEDCs expressed high levels of CCR5, CXCR4, and CD4 which are all key entry receptors for HIV infection. Due to their recent identification the ontogeny and functionality of these cells is yet to be further characterized in humans.

#### Inflammatory Dendritic Epidermal Cells (IDECs)

Work done by Wollenberg et al. in the late 1990's identified a novel epidermal DC subset present within inflamed skin which they termed inflammatory dendritic epidermal cells (IDECs) (124) (Figure 2A). Using biopsies from inflammatory skin conditions including atopic dermatitis and eczema, they phenotyped these novel cells as CD1a^+^ Langerin^−^, lacking Birbeck granules and having increased FcεR expression, distinguishing them clearly from the LCs which were also present (124, 125). They have also been shown to express CD11b, CD11c, MR, and DC-SIGN (CD209).

Based on the lack of Birbeck granule and surface molecule expression it is possible that these cells are similar to VEDCs, however, despite these cells being characterized two decades ago, limited work has been done to determine their ontogeny or function. While their function still remains unknown, functional differences have been reported between IDECs and LCs, with the former showing no signs of dendritic extensions through tight junctions to process antigens unlike what has been shown by LCs (126). Furthermore, both IDECs and LCs within atopic dermatitis skin have been shown to have markedly lower TLR2 expression compared to LCs in healthy skin, while IDECs and not LCs have markedly higher levels of the maturation marker CD83 and MHC class I and class II molecules (127).

### Dermal/Lamina Propria Dendritic Cells

The dendritic cells in the underlying dermis/*lamina propria* are also of importance within the context of HIV infection. If the physical barrier of the epidermis is compromised such as by physical trauma or inflammation, HIV can come into direct contact with these cells. While a lot of research has focused on skin dermal DCs, recent studies in mucosal sites such as the gut and female genital tract have determined new classifications of the cells that reside there.

#### Tissue cDC1

As previously described in blood, cDC1s in both lymphoid and non-lymphoid tissues carry a similar phenotype suggesting a precursor-progeny relationship (103). Here they are characterized by their high expression of CD141, moderate levels of Clec9A, XCR1, and CADM1 and low to negative levels of CD11c, CD14, CD1c, CD11b, and SIRPα (103, 128). They have continuously been shown to have an increased capacity for cross presentation of antigens compared to other dermal DC subsets which is further increased with TLR3 stimulation (103). Tissue cDC1s produce high levels of TNF-α and CXCL10 following stimulation, while showing limited production of IL-1, IL-6, IL-8, IL-10, IL-12, and IL-23 (103) and have been shown to weakly promote a Treg response in intestinal tissue (128).

Previously in the literature, cDC1s have been identified by CD141 and CADM1 expression and lower expression of CD11c. However, Botting et al. recently showed that all skin mononuclear phagocytes express CADM1, and during the process of spontaneous migration cDC2s upregulate CD141 and cDC1s upregulate CD11c (104). Thus, cDC1 are almost impossible to confidently identify using this method of isolation. These findings further emphasize the importance of isolation methods to ensure correct identification of DC subsets in tissue and when analysing data in the literature.

#### Tissue cDC2

cDC2s in tissue express more C-type lectins (CLRs) than their blood counterparts (e.g., Mannose receptor), and have a more activated phenotype expressing higher levels of the maturation markers CD80, CD83 and CD86 (103, 128, 129). In tissue, cDC2s express CD1a, CD1c, CD11c, and SIRPα (49, 74). Similar to epidermal LCs, cDC2s have also been shown to be DC-SIGN^−^, which helped support the idea that DC-SIGN is not a pan DC marker (130). The absence of Birbeck granules as well as higher CD11c and CD11b expression on cDC2s in the dermis helps distinguish these cells from LCs (50, 111, 131). They express TLRs 1-9 (50, 132) while producing a range of cytokines including IL-1β, IL-6, IL-8, IL-10, IL-23, CXCL-10, and TNF-α (103) and have shown to stimulate a Th17 response in intestine (128).

Recently, a Langerin-expressing subset of cDC2s were identified in the dermis of the skin distinct from LCs (50, 111). These cells are phenotypically and genotypically related to cDC2s expressing moderate levels of CD11b, CD11c, and CD13 which are all absent or lowly expressed by LCs, whilst also expressing lower levels of CD1a and Langerin compared to LCs (50, 111). Further distinguishing them from LCs that have migrated from the epidermis is their replacement kinetics (111) and presence in sites where LCs are not found, including the lungs (111) and intestine (133). Further investigation is needed to determine if this Langerin expressing subset differs in its functionality compared to Langerin^−^ cDC2s.

#### CD14-Expressing Cells

Cells expressing CD14 make up a large proportion of the mononuclear phagocyte population in human tissues. Until recently it was thought this population was made up of two distinct subsets, tissue resident autofluorescent macrophages and non-autofluorescent DCs (134). In 2014 McGovern et al. showed that the non-autofluorescent CD14^+^ cells were actually monocyte-derived macrophages (135). Replacement kinetics and transcriptomic studies suggest a precursor-progeny relationship between these cells and CD14^+^ monocytes (135). Furthermore, McGovern et al. showed these cells have limited induction of naïve T cells compared to tissue cDC2s, while being strong stimulators of memory CD4^+^ T cells comparable to both tissue cDC2s and macrophages (135). However, it is of note that skin CD14^+^ cells can be split into two subsets according to CD1c expression and it is the CD14^+^ CD1c^−^ cells that McGovern defined as monocyte-derived macrophages. It is still unclear whether the CD14^+^ CD1c^+^ cells are more macrophage or dendritic cell like. Interestingly, it is CD14-expressing cells only that express DC-SIGN, which was previously believed to be a DC marker. Thus, DC-SIGN is likely to in fact be a marker of macrophages.

Recent publications investigating CD14-expressing cells in the mucosa of the gut have shown this cell compartment to be made up of four distinct macrophage populations, termed Mf1-4 (136) (Figure 2C). These four subsets cans be distinguished by their expression of CD11c, HLA-DR and CD11b: Mf1 and Mf2 are CD11c^+^ with the former having lower expression of HLA-DR compared to the latter, while Mf3 and Mf4 are CD11c^−^ with the former being CD11b^−^ and the latter CD11b^+^. Furthermore, from the transcriptomic and replacement kinetic analysis of these four subsets, Bujko et al. suggested that these cells are derived from incoming peripheral blood monocytes which progressively differentiate to Mf1s and Mf2s as an intermediate before further differentiation to either Mf3s or Mf4s. Whether this characterisation is relevant in other non-lymphoid tissues where these cells have been more thoroughly studied, such as the skin or mucosal tissue such as the cervix and vagina, has yet to be determined.

### Intestinal Dendritic Cells

Using CD103 and SIRPα, Watchmaker et al. characterized three distinct DC populations within healthy human small intestine, each of which could be related to previously studied human blood DCs and mouse tissue DCs (128) (Figure 2C). Within this tissue the dual positive population (CD103^+^ SIRPα^+^) was the dominant subset and was shown to be closely related to blood cDC2s, sharing common transcription factors IRF4 and PR domain zinc finger protein 1 (PRDM1). The single positive CD103^+^ SIRPα^−^ population was shown to be closely related to blood cDC1s with conserved expression of IRF8 and B-cell lymphoma 6 (BCL6), and the CD103^−^ SIRPα^+^ subset shared common transcripts with CD14^+^ monocytes. Watchmaker et al. then went on to show distinct differences in the functionality of these cells, with the dual positive population and the CD103^−^ SIRPα^+^ subset showing much higher levels of T cell proliferation compared to the single positive CD103^+^ population. The dual positive population also induced significantly higher levels of Foxp3 expression in these proliferating cells suggestive of a Treg phenotype. Furthermore, it was shown that the dual positive and single positive CD103^+^ population induced higher levels of IL-17-producing Th17 cells while the CD103^−^ SIRPα^+^ subset produced Th1 interferon-γ-producing cells.

More recently these intestinal DCs have been investigated in more detail, confirming the relationships with their blood counterparts suggested by Watchmaker et al. (128) but also highlighting the importance of using CD14 as a marker to differentiate monocyte-derived cells (CD14^+^) from bona-fide DCs (CD14^−^), suggesting that previous analysis of these cells likely included a mix of the two (137). This was underscored by extensive RNA sequencing analysis which showed both the CD14^+/lo^ dual positive CD103^+^ SIRPα^+^ and CD14^+/lo^ single positive CD103^+^ cells had monocyte lineages, clustering with subsets of the CD14^+^ Mfs described by Bujko et al. whereas the CD14^−^ subsets aligned with a bona-fide cDC2 signature. This was further supported by their antigen uptake abilities, with the CD14^+/low^ subsets showing increased ability to take up *Escherichia coli* by PHrodo analysis compared to the CD14^−^ populations, consistent with previous findings that show human small intestine macrophages are more efficient at antigen uptake than DCs (136). Finally, the migratory kinetics of these cell were assessed, with the dual positive population showing the highest rate of CCR7-dependent migration out of the tissue, whereas the single positive population had the lowest. Richter et al. hypothesized that these results suggested the single positive CD103^+^ population was therefore made up of a larger proportion of monocyte-related cells compared to the double positive population.

### Inflammatory Dendritic Cells

In inflammatory conditions, a number of distinct tissue DCs have been identified including CD16^−^ CD1c^+^ DCs (138), TNF-α and inducible nitric oxide synthase (iNOS)-producing DCs (TIP-DCs) (139, 140) and Slan^+^ DCs (141, 142) (Figure 2A). CD16^−^ CD1c^+^ DCs have been described in inflammatory conditions including within synovial fluid of arthritis patients and tumor ascites, expressing CD14, CD11c, CD1a, CD11b, MR, SIRPα, and FcεR1. These cells were shown to have a DC like morphology and a transcriptomic approach showed this subset to be distinct from known DC subsets while still sharing common gene signatures with *in vitro* monocyte-derived DCs. Furthermore, these cells were able to produce a high Th17 response in naïve CD4^+^ memory T cells. With their CD14^−^ CD1c^−^ phenotype both TIP-DCs and Slan^+^ DCs can be distinguished from the CD16^−^ BDCA^+^ DCs. Both TIP-DCs and Slan^+^ DCs have been identified in psoriatic skin, while Slan^+^ cells have also been identified in *lupus erythematosus* (141), steady state skin (143), and tonsil (144) and been shown to produce a range of cytokines upon stimulation including IL-6, IL-23, TNF-α, IL-12, and IL-1β (53, 141, 142, 144). Whether any of these cell subsets are present in inflamed tissue of the human anorectal tract is yet to be determined.

## The Role of Dendritic Cells in HIV Infection

**Figure 3:**
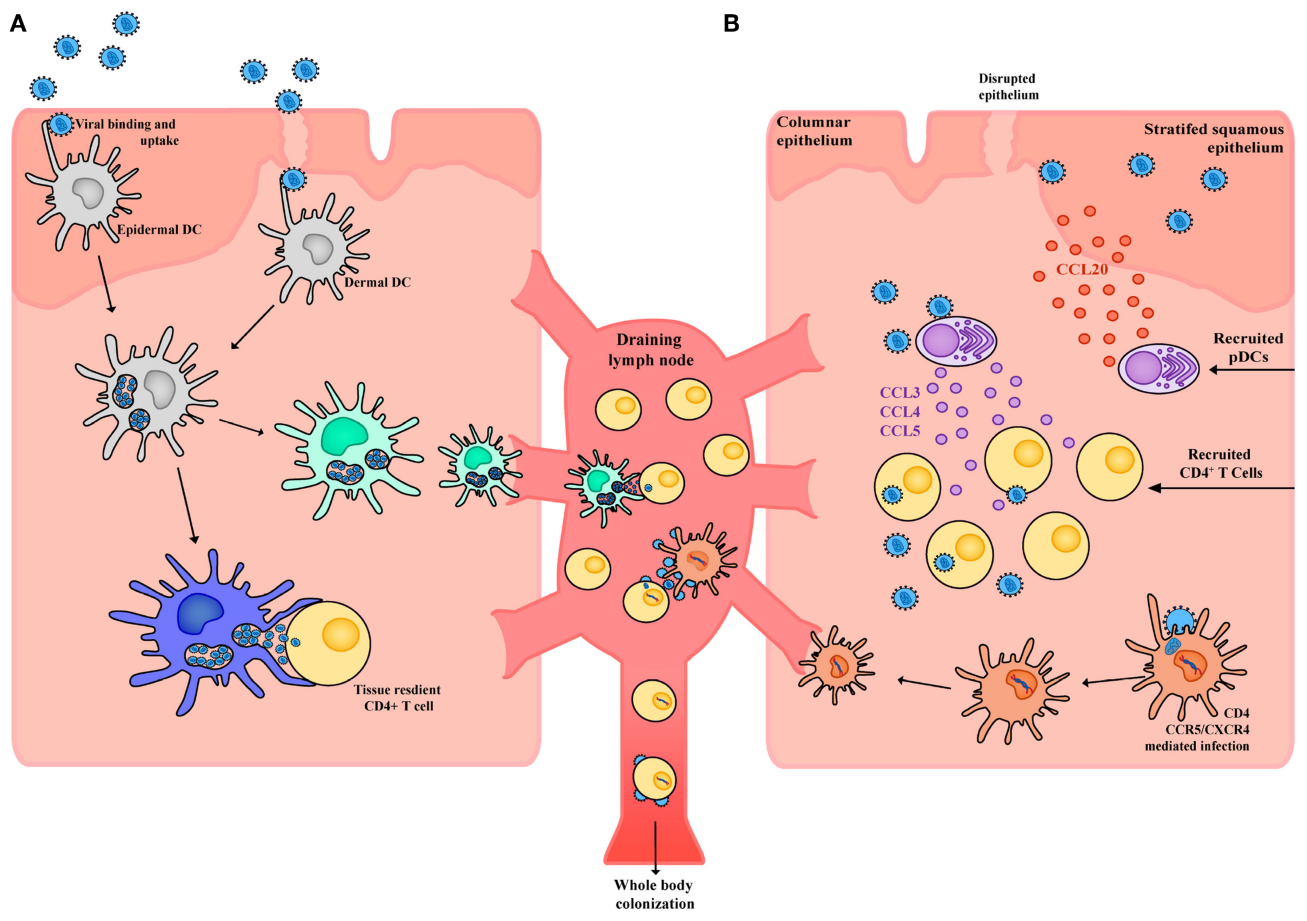
Schematic of HIV interactions with DCs in tissue. **(A)** Within the stratified squamous epithelium of skin and type II mucosa epidermal DCs pick up incoming HIV virions potentially through a wide array of receptors including CLRs, syndecans, and CD169. Virus is taken up into open vesicular compartments that are more extensively formed in proinflammatory conditions. DCs harboring virus (yet not productively infected) can directly and immediately transfer the virus to tissue resident CD4^+^ T cells or can migrate to the draining lymph node and transfer the virus to CD4^+^ T cells that reside there. For the latter to occur, maturation of DC subsets will increase the half-life of HIV to enable efficient transfer. **(B)** Whilst in immature DCs, the half-life of captured HIV would not be conducive to transfer after migration in draining lymph nodes. Given immature DCs are susceptible to infection, latter viral transfer may proceed if they are infected. From here the DC migrates to the draining lymph node and productively infects CD4^+^ T cells. Of note, whilst DCs may not be highly susceptible to HIV infection, low frequency infections can still lead to robust viral transfer when in contact with CD4^+^ T cells. This is analogous to communication of the immune response by DCs, where only limiting numbers can still mediate a productive immunological outcome. Finally, in response to HIV infection epithelial cells can produce CCL20 which drives the migration of pDCs into the tissue. These pDCs in turn produce high levels of CCL3, CCL4, and CCL5 which drive migration of CD4^+^ T cells into the tissue, increases CCR5 expression and in doing so further facilitates local HIV infection and spread.

When a cell is identified as a viral substrate, often a whole continuum of phenotypes and subsets is branded in a similar manner. Whilst it is clear that CD4^+^ T cells are the primary substrate of HIV, not all subsets are equally permissive to infection and each subset can have very different outcomes when infected. The same can also be applied to the unique continuum of mononuclear phagocytes in the form of their phenotype and what specific subset they have differentiated into. HIV interactions with DCs can rarely be described as one size fits all. Our attempts as a field to do this with the identification of DC-SIGN as a HIV receptor on *in vitro* monocyte-derived DCs is an example where we lose the bigger picture in the context of DC-HIV interactions (145, 146). Rather than one receptor-HIV envelope interaction, the interactions of HIV with DCs is complicated and informs us of a sentinel immune network that is built for distinct roles *in vivo*—that is, there is a division of labor and no one DC subset behaves the same (130, 147). As a consequence, no single DC subset interacts with HIV in an identical manner. Whilst the first contact of HIV with the virus is complicated, so too are the outcomes. Over time, many investigators have staked their position in one of two camps: the first is that DC subsets need to become infected with HIV to mediate viral spread or the second, where DC subsets simply carry HIV to enable safe passage and transfer to a secondary lymph node where a contacting resident CD4^+^ T cell is the unfortunate recipient (Figure 3). In reality, both camps are correct, yet depending on the DC subset there can indeed be bias with which camp the observation sits in. As a field, we need to be open to both and appreciate the continuum of outcomes, however complex they turn out to be.

### Historical Perspective of Dendritic Cells in HIV Infection

From here on, it is important we outline the initial seminal contributions of how DCs interact with HIV and how each DC phenotype (often influenced by DC isolation) plays a significant role in past observations. We will then fast forward to outline how exponentially expanding/powerful technologies have now given us further insights into how rich the DC subset landscape is and importantly how this landscape sits *in vivo* and importantly in the context of HIV pathogenesis.

#### The Dawn of the Interface of Dendritic Cell and HIV Biology

Soon after the discovery of HIV as the causative agent of Acquired Immunodefiency Syndrome (AIDS) were early and successive reports of HIV antigens within lymph nodes of patients with persistent generalized lymphadenopathy (148, 149). In this setting, virus was observed in the context of germinal centers and concentrated in association with follicular dendritic cells (fDC). Whilst isolation and mechanistic dissection of the role of fDCs was not initially possible, later studies on HIV-fDC dynamics highlighted their role in endocytic capture and preservation of HIV in the germinal centers of lymph nodes (150). Thus, instead of their role in the preservation of native antigens for B cell presentation, fDCs were saturated with HIV virions and facilitating the preservation of virus. Shortly following immunopathological studies of lymph nodes was the observation of HIV in association with Langerhans cells in skin biopsies of patients clinically presenting with AIDS (151). Through the use of electron microscopy, these early studies could readily pick up features of Langerhans cells through the presence of Birbeck granules (151), with virus at times in association with them (152) and evidence of viral budding and cytopathicity (151). Whilst more recent work has resolved how LCs interact with HIV, it was clear that these primary observations pointed to LCs being directly infected with HIV (as evidenced by viral budding) and capturing HIV in compartments enriched in receptors such as the CLR Langerin (as evidenced by the presence of Birbeck granules). Whilst many of these early seminal studies pointed to a role of several dendritic cell subsets in HIV pathogenesis, lack of dendritic cell markers and difficulties faced in their isolation for *in vitro* studies limited the mechanistic understanding of how each DC subset was encountering HIV.

#### Understanding HIV-DC Interactions Through the Early Studies of Blood Dendritic Cells

Work in the laboratory headed by Knight and Patterson (153–155) would start the journey on a preliminary understanding of how HIV can interact with DC subsets, using DCs isolated from blood. So as not to confuse the observations of early studies on blood dendritic cell subsets and more contemporary studies, it is important to outline early DC isolation techniques as they often involved *in vitro* culture steps that led to distinctly different phenotypes, as opposed to the freshly isolated DC subsets we now have the power to isolate from human blood or from tissue (104). In these early isolation methods, short-term culture of PBMCs isolated by Ficoll gradients would primarily deplete monocytes by adherence, and through subsequent and often intricate metrizamide gradient separations, buoyant blood dendritic cell populations could be isolated with purity assessed by the lamellipodia/veils on what we now recognize as partially mature DCs (maturation often resulting from the *in vitro* culture). During the same time, work in the Steinman laboratory led by Paul Cameron and colleagues further combined early immuno-depletion methods with similar gradient based enrichment but for the first time included stringent selection of dendritic cells through fluorescence-based sorting for cells without immune lineage markers; lineage being defined at the time as abundant immune markers that could readily be used to detect monocytes (CD14), NK cells (CD16), B cells (CD19), and T cells (CD3).

However, the outcome of these early studies was often not consistent across laboratories, with the work led by Knight and Patterson highlighting the infection of dendritic cells whilst observations by Cameron et al. culminated in the seminal observation that DCs primarily capture HIV without being infected and in such an efficient manner that it produces vigorous cytopathic infection upon coculture of CD4^+^ T cells with HIV-exposed DCs (156). This latter observation is what is now referred to as infection “*in trans*.” Although the term “*in trans*” was not coined by Cameron and Steinman, they provided the first seminal evidence it could occur by culturing lineage^−^ murine DCs and observing they could be used to capture and transfer virus to human CD4^+^ T cells in a manner equivalent to human blood-derived DCs (156). Given the knowledge that murine cells are refractory to HIV infection, this was the most stringent example of DCs binding, capturing and later transferring virions to recipient CD4^+^ T cells without becoming infected themselves. These differing observations in the research by Patterson and Knight vs. that of Steinman and Cameron brought forward the first indications that not all DC-HIV interactions were equal. Indeed, across both laboratories, the intricate isolation conditions led to each lab's DCs exhibiting different end-stage phenotypes. For Patterson and Knight, a partially mature DC population that could sustain HIV infection persisted, whilst for Steinman and Cameron, the stringent sorting of blood DCs resulted in the isolation of a purer and more mature DC population. What we now know is that the primary discrepancy in the infection of DCs vs. DCs simply carrying virus is a combination of their maturation state (157, 158) and the source of viral inocula used for infection (159).

#### Early Observations With Skin-Derived DCs

Shortly following the seminal observations by Steinman and Cameron were observations by Melissa Pope and Steinman of resident dendritic cells that emigrated from skin which could also induce a vigorous cytopathic infection in T cells when exposed to virus (160). Unlike the early work by Cameron that highlighted the first *in trans* transfer of HIV to T cells, the mechanism of DC-HIV capture and transfer in skin DCs was initially unclear. Follow up studies led by Pope et al. demonstrated that transfer of virus from skin DCs required a low level of infection to ensure efficient viral spread throughout the DC-T cell culture (161). However, like the work that initially dissected mechanisms in blood dendritic cells, it must be noted that emigrating DCs have a phenotype that diverges from their resident immature counterpart. In addition, as we will discuss in more contemporary studies on DC-HIV interactions, the DCs used in these studies would have been comprised of multiple populations of tissue resident DCs. Whilst isolation of emigrating DCs from skin was the method initially used to dissect DC-HIV interactions, follow up studies by Reece et al. (162) and Kawamura et al. (163) would take this closer to an *in vivo* snapshot through the inoculation and infection of Langerhans cells in skin, ultimately proving that transfer of virus from LCs exposed to HIV *in situ* was closely linked to their ability to become infected by the virus (162).

#### The Introduction of the Monocyte-Derived Dendritic Cell Model

The early observations of DC-HIV interactions were largely constrained by a limited availability of DCs. Often representing <1% of the tissue or blood immune population, isolation of DCs and infection were considered “herculean” tasks. However, an alternative to this limitation was achieved through the identification by Sallusto et al. (77) and Romani et al. (164) in 1994 that a combination of GM-CSF and IL-4 could differentiate abundant CD14^+^ monocytes into cells that closely resembled DCs found *in vivo*, which could be further matured into a terminally differentiated mature DC using pro-inflammatory cytokines. Whilst many DC “purists” took observations of MDDC-HIV interaction with caution, many studies using this model could recapitulate the results observed using primary DC subsets, namely that MDDCs could be infected (158), could transfer virus once infected, and could also transfer virus independent of infection (158, 165). Furthermore, it was evident in the seminal work by Blauvelt et al., that DCs bound virus in a very different manner to CD4^+^ T cells (165)—following this work was the re-identification of a HIV gp120-binding CLR (DC-SIGN) expressed at high levels on MDDCs (145) [initially isolated from placental cDNA by Curtis et al. (166)]. Now named DC-SIGN from its description on DCs *in vitro* and ability to facilitate integrin binding during DC-T cell interactions, many of the HIV-DC interactions were initially assigned to this HIV-lectin interaction.

#### In vivo/Real DC Subsets and the Complexity Beyond the MDDC Model

Shortly after the characterization of the C-Type lectin pathway in MDDCs was the journey into understanding how each DC subset uniquely bound and interacted with HIV. Whilst it was clear that blood dendritic cells could bind and efficiently transfer HIV to CD4^+^ T cells, it was readily assumed they expressed DC-SIGN. Subsequent studies not only highlighted this was not the case but further demonstrated that each DC subset had its own unique repertoire of binding receptors, some of which would enable transfer of HIV whilst other would mediate their infection (130, 167–169). From here onwards, we will refer to recent contemporary studies that have mapped the diverse landscape of dendritic cells and how they interact with HIV.

### Contemporary Understanding of DCs in HIV Infection

Although it has been shown that the majority of HIV binding and uptake in DCs occurs through CLRs (158), this process is highly subset-dependent (not occurring in freshly isolated DCs derived directly from blood) and can be heavily influenced by the maturation state of the DC (130, 157, 158). The same can be said for infection following CD4 and CCR5-dependent entry, which varies across DC subsets and is also influenced by maturation (158). The source and purity of viral inocula also plays a significant role in the outcome of infection in DCs. While many still make the commentary that DCs cannot be infected or that HIV has evolved not to infect DCs, there are several points that should be noted. Firstly, as outlined above, the earliest studies of DC-HIV interactions could observe infection *in vivo*. Secondly, the concept that lentiviral restriction factors such as SAM domain and HD domain-containing protein 1 (SAMHD1) do not enable HIV infection solely in DCs is not correct as lentiviral restriction is also observed across many susceptible HIV targets including T cells and macrophages (170, 171). Finally, maturation of DCs can lead to significant blocks in HIV infection and culture/inoculation conditions that favor maturation of DC subsets will likely not reveal any HIV infection. Indeed, the continuum of DC phenotypes that influence how HIV is captured by DCs, through to if a DC subset can be infected, also modulates its potential to disseminate and transfer virus following exposure to HIV.

Transfer of HIV from DCs to CD4^+^ T cells appears occurs in two stages, as determined by studies in both *in vitro* MDDCs (158, 172) and *ex vivo* LCs (173). First-phase transfer (within 24 h) relies on transient uptake of virus through pattern recognition receptors (such as Langerin and other CLRs) which either leads to proteolytic degradation of virus in the endosome or immediate transfer across the virological synapse (174, 175) (Figure 3a). Alternatively, second-phase transfer occurs over a longer phase (around 96 h) and is mediated by initial CD4/CCR5 mediated neutral fusion at the DC membrane and productive replication of HIV (158, 176, 177) (Figure 3b). *De novo* virus can subsequently be transferred to CD4^+^ T cells across the virological synapse, which uses adhesion factors such as intercellular adhesion molecular 1 (ICAM-1) to stabilize DC-T cell contacts (178), with infection being established more effectively than direct infection by free virus (156).

The mechanism for uptake of virus in the first-phase is complex—whilst initial studies supported recirculation of virus from endosome networks (179), later studies have revealed the virus for transfer is accessible to the surface (180), but compartmentalized in CD81-positive open membrane invaginations that appear to resemble endosome like compartments (181). Curiously, whilst immature and mature DCs can both mediate this type of transfer, it is clear that mature DCs can sustain larger reservoirs of virus for first-phase transfer/*trans* infection (157, 158). This *in trans* infection *trans* model has received much attention as it can be applied across many DC subsets and does not rely on the intricacies of studying low level infection of DCs over time. However, two concepts should be emphasized for this form of transfer—firstly, in immature DCs this phenotype is short-lived and independent of the DC subset. For instance, immature Langerhans cells and MDDCs equally bind (and destroy) many incoming virions over periods of ~24 h (158, 182). Upon maturation, DCs can increase the half-life of bound and trafficked virus, but eventual viral decay and leading to an inability to HIV transfer proceeds (158). Secondly, this process of transfer does not discriminate between strains of HIV that utilize the CCR5 co-receptor or CXCR4 co-receptor for entry (known as R5 and X4 viruses, respectively) whereas whilst it is clear that there is a distinction between them *in vivo* (183). As such, while only a small percentage of DCs become productively infected and can undergo second-phase transfer compared to primary T cells, this still represents a route of transfer as well as a potential mechanism for latent infection of T cells. In addition, productive infection ensures second-phase transfer of virus has a half-life far longer than virus that is simply bound or trafficking through a cell, and in that context provides greater transfer potential. In addition, many studies have observed infected DCs have superior viral transfer capacities than CD4^+^ T cells and can often efficiently spread infection even in limiting numbers (161, 184).

### In vivo: First Contact

Due to their anatomical localization in the outer epidermis of the human anogenital tract, LCs are thought to be the first cells to capture HIV upon sexual transmission (185–187) and therefore their interactions with HIV have been extensively studied. A large amount of this work conducted on LCs has used cells isolated from skin explants rather than anogenital tissue as they are much easier to access and are much larger in size, allowing for significantly higher cell yields and thus enables functional assays to be performed. Early studies have consistently shown LCs to be infected with HIV both *in vivo* (188–190) and *ex vivo* (173, 191–194), suggesting an important role for these cells in transmission. Furthermore Nasr et al. showed uptake and transfer of the virus to CD4^+^ T cells from *ex vivo* LCs occurred in two phases as described above (173). Furthermore, they went on to show this was mediated by the CLR Langerin and could be efficiently blocked using an anti-Langerin monoclonal antibody or soluble Langerin, highlighting the differences in each specific DC subset and their CLR profiles they use to interact with HIV (173).

LCs have also been investigated in penile tissue (195–200), cervix (196), and vagina (201, 202) as a target cell for HIV infection. It has been shown that within the male genital tract (MGT) there is an increase of LCs found in the glans penis compared to both inner and outer foreskin (195), while other studies have shown increased HIV co-receptor CCR5 expression on LCs in the inner foreskin compared to the outer foreskin (203). Explants on MGT tissue with HIV have shown penetration of the virus to depths where LCs are abundant, particularly within the inner foreskin and uncircumcised penis (197). These findings were consistent with macaque model work performed co-currently, suggesting these explant models are representative of *in vivo* observations. Furthermore, explant and *in vitro* modeling has shown cell-associated virus translocating through inner foreskin keratinocytes can be sampled by LCs which migrate toward the apical epithelium in response to the invading pathogen (199, 200). These LCs quickly internalize HIV and *trans* infect epidermal CD4^+^ T cells, with increased T cell-LC conjugates following HIV infection confirmed by flow cytometry. Exacerbating this infiltration of HIV via LCs is an increased production of CCL5 (RANTES) by these LCs, driving the migration of CD4^+^ T cells into the epidermis (198). In *ex vivo* models vagina LCs are able to endocytose HIV (202) and then proceed to *trans* infect CD4^+^ T cells without showing signs of being productively infected themselves (201).

The recent identification by Pena Cruz et al. of CD1a^+^ VEDCs, which express high levels of HIV receptors CD4, CCR5, and CXCR4, also require further investigation into what role these cells play in the process of transmission. In their study, only viruses which use the CCR5 co-receptor (known as R5 viruses) and not those which use the CXCR4 co-receptor (known as X4 viruses) were found to replicate efficiently within these cells *ex vivo* (121). However, it was shown that the decreased replication with X4 virus was not due to decreased fusion of the virus to the CD1a^+^ VEDCs, with levels of R5 and X4 viruses binding and fusing at comparable levels. While both strains showed signs of integration and reverse transcription, R5-enveloped virus had significantly higher integration and reverse transcription levels compared to X4 virus, which was shown to be influenced by the HIV restriction factor SAMHD1. Finally, *in vivo* work showed CD1a^+^ VEDCs harbored high levels HIV DNA in virologically suppressed women and thus these novel cells may represent a potential latent reservoir for HIV within vaginal tissue.

While a lot of work has been done on LCs and their interactions with HIV, dermal, and *lamina propria* DCs have not been as extensively investigated, particularly within the anorectal tract. However, studies have been able to show intestinal DCs taking up virus and *trans* infecting blood and intestinal CD4^+^ T cells (204–206). Cavarelli et al. used both an *ex vivo* and *in vitro* model, to show that intestinal DCs (defined as CD11c^+^ CD68^−^ to exclude macrophages) migrate toward R5 virus, extending dendrites through the intestinal epithelium to capture R5-HIV (204). This was driven by the R5-envelope itself engaging with cellular CCR5, with no evidence of CCR5-binding chemokines present in these models as a driving force for this migration. Furthermore, these cells were shown to then efficiently *trans* infect target CD4^+^ T cells. This *trans* infection to CD4^+^ T cells has also been seen using small intestine explants (206) as well as primary rectal mononuclear cells (205). However, with the ever-changing re-classification of DC subsets, particularly within the human intestine, it is unclear what subset these cells represent and how these findings translate *in vivo*.

*Lamina propria* myeloid dendritic cells of the vagina (207–210) and cervix (209–211) have also been shown to capture and *trans* infect HIV efficiently, with no significant differences found between different anatomical sites of the female reproductive tract (FRT) (209, 210). Using vaginal explant models, Shen et al. showed HIV^+^ cells that had migrated out of the mucosa after 2 h were of a myeloid DC phenotype expressing CD11c and CD13, while macrophages (CD11c^−^, CD13^+^) and lymphocytes (CD3^+^) were HIV^−^ (207, 208). Moreover, cervical myeloid dendritic cells (CD14^+^ CD11c^+^) have been shown to efficiently take up R5-HIV strain, more so than lymphocytes (CD4^+/low^) and macrophages (CD14^+^ CD11c^−^) but do not show signs of productive infection at a later time point, unlike the lymphocyte population (211). Following HIV infection of tissue of the FRT, *lamina propria* DCs show increased secretion of CCL2, CCL3 and CCL4 as well as a moderate increase in IL-8, while there is no difference in secretion of pro-inflammatory cytokines including IL-6, IL-1β, and TNF-α (209, 210). Furthermore, these cells show a short-lived increase in secreted antimicrobials including elafin, CCL5 and secretory leukocyte peptidase inhibitor (SLPI) following infection. It must be noted however that the CD14^+^ CD11c^+^ cells looked at within the mucosal tissue in these studies may in fact be a subset of macrophage as has been recently identified in the small intestine (136) and therefore will have to be characterized further.

While it is quite well documented that anogenital inflammation is a major risk factor for the sexual transmission of HIV (212–214), it is still not well characterized what DC subsets are present in the human anogenital tract in these conditions and what role these cells may play. While a number of DC subsets have been identified in inflammatory conditions in other tissues, further work needs to be done to confirm these cells reside within the tissues HIV encounters upon sexual transmission, and subsequently how these cells interact with HIV. However, it has been extensively shown that upon inflammation there is a large influx of HIV target cells including DCs as well as CD4^+^ T cells and macrophages, suggesting that perhaps it is not the occurrence of novel subsets of DCs but rather an increased density of target cells for HIV to interact with.

### The Role of pDCs and the Innate Response During HIV Transmission and Infection

Although plasmacytoid DCs are not found constitutively in peripheral tissues, they are recruited to sites of viral exposure and inflammation through engagement of various chemokine receptors, particularly CCR2, CCR5, CCR6, CCR9, CCR10, CXCR1, and CXCR3 (215–221). Within the context of HIV, Shang et al. have demonstrated that CCL3, CCL20, and CXCL8 are produced by cervical epithelium within 24 h of simian immunodeficiency virus (SIV) infection—CCL20 chemotactically recruits CCR6^+^ pDCs to the underlying endocervical mucosa, with CCR5^+^, CCR6^+^, CXCR1^+^, and CXCR2^+^ cervical macrophages also attracted through this mucosal signaling axis (222). The cervical macrophages in turn produce CCL3, CCL5, CXCL8, and CXCL10, further recruiting CCR5^+^, CXCR1^+^, and CXCR3^+^ pDCs to the mucosa. As such, a chemotactic “sink” at the site of SIV/HIV exposure leads to the rapid recruitment of pDCs within 1–2 days post infection (222, 223), which then exert a large influence on the course of early HIV infection.

Given their well-documented production of IFN-I in response to HIV (particularly IFN-α1/13, 2, 5, 8, and 14, IFN-β and possibly IFN-ω) (224–229), pDCs have typically been associated with early antiviral responses that limit early viral replication and dissemination. Although HIV completely inhibits the IRF3-mediated IFN-I response in myeloid DCs, macrophages and CD4^+^ T cells upon uptake or infection (230–235), pDCs constitutively express high levels of IRF7 and upon sensing endosomal HIV ssRNA through TLR7, IFN-I production is rapidly and potently induced (225). Consequently, the IFN response during infection appears to be primarily dictated by pDCs, particularly during acute HIV/SIV infection (223, 236, 237), despite their low frequency in circulation (~0.001%) (9, 10, 238). The effects of early IFN production on HIV infection appear to be mostly protective and are best observed in rhesus macaque (*Macaca mulatta)* studies where disease outcome can be more easily assessed. Blockade of the IFN-I receptor for 3 days following acute rectal SIV infection accelerated depletion of CD4^+^ T cells and resulted in an expansion of the viral reservoir due to abrogation of interferon-stimulated gene (ISG) induction, eventually leading to increased and more rapid progression to AIDS (239). Similar results have also been reported upon blockade of IFN-α blockade prior to intravenous SIV infection (240). Meanwhile, exogenous administration of IFN-α2 prior to intrarectal SIV inoculation delayed systemic infection, upregulating the expression of ISGs and necessitating up to four additional challenges for transmission (239). This apparent protection conferred by IFN-I during early infection is also corroborated by a single topical application of IFN-β being sufficient to protect a majority of vaginal simian-human immunodeficiency virus (SHIV)-inoculated macaques from infection (241). Studies of the transmitted/founder (TF) virus that singularly establishes infection also indicate that IFN-resistance is a key trait in most of these viruses, suggesting that IFN-I are amongst the most important selective pressures exerted by the host during transmission (242, 243).

Interestingly, IFN-Is have also been reported to induce T cell activation—topical vaginal IFN-β application led to an increased density of vaginal HLA-DR^+^ and CCR5^+^ CD4^+^ T cells in rhesus macaques and induced a highly pro-inflammatory state with increased expression of CXCL1/10/11, CCL7/8/23, IL-1β, IL-6, IL-8, TNF-α, and IFN-γ within the female reproductive tract (241). IFN-I produced by pDCs has also been shown to induce CD69 and CD38 expression on peripheral blood CD4^+^ T cells (244). Altogether, it appears that early IFN-I production exerts an antiviral effect sufficient enough to play an overall protective function despite increasing the susceptibility of local HIV target cells to infection. pDCs are also known to produce IFN-III in response to HIV, which can exert antiviral effects in infected CD4^+^ T cell (245, 246) how this formally contributes to HIV transmission and early infection is not known.

In addition to IFN-I, pDCs are known to produce other soluble mediators when exposed to HIV inocula including TNF-α, IL-6, IL-13, and IL-12 (238). Both IFN-α and TNF-α have both been shown to mature DCs (247, 248) which may have important consequences for the spread of HIV infection *in trans* by (1) enhancing DC-mediated viral transfer to CD4+ T cells at initial mucosal sites, (2) accelerating cellular transport of virus to other lymphoid compartments, and (3) increasing the half-life of virions bound and trafficking through DC (158). In addition, TNF-α is known to upregulate the expression of the transcription factor NF-kB, which is required for HIV proviral transcription in CD4^+^ T cells (249), and so likely accelerates the lytic HIV lifecycle and CD4^+^ T cell depletion during infection (250). However, the *in vivo* relevance of these pro-inflammatory effects of pDCs is unclear—during acute vaginal infection in rhesus macaques, SIV-infected CD4^+^ T cells at the vaginal mucosa appear to predominantly reside in a resting state, typically not expressing CD25 or other T cell activation markers (personal communication with Ashley Haase). Beyond pro-inflammatory cytokines, pDCs also play a key role in recruiting CD4^+^ T cells to mucosal sites of infection through the production of the inflammatory CCR5-binding chemokines CCL3, CCL4, and CCL5 (222, 223) (Figure 3b). The production of these chemokines leads to an influx of CCR5^+^ CD4^+^ T cells to sites of infection and fuels the expansion of infected founder CD4^+^ populations during acute vaginal SIV infection, thereby allowing foothold infection to be established. Intravaginal application of glycerol monolaurate inhibits secretion of CCL20 and protects against repeated high-dose vaginal SIV challenge, suggesting that pDCs are key cells that underpin the permissive chemotactic and inflammatory milieu during successful transmission events (223). Of note, aside from pDCs and macrophages, other leukocytes such as B cells and neutrophils do not appear to accumulate in SIV-infected cervical tissue or co-localize with clusters of SIV-RNA^+^ or CD4^+^ T cells (222, 223), suggesting CD4^+^ T cell recruitment is the main chemotactic role pDCs have during infection.

In addition to these multifaceted roles during early infection, pDCs have also drawn interest due to their ability to reactivate virus from latently-infected cells, having been associated with decreases in CD4^+^ T cell proviral load in suppressed patients upon combined latency-reversal agent and TLR agonist treatment (251–255). IFN-I signaling can lead to the induction of IRF1 (256) which, like NF-κB, promotes transcription of HIV proviral DNA. pDCs have also been reported to prevent the establishment of HIV latency in primary resting CD4^+^ T cells in an IFN-α-mediated process (257, 258). The deleterious role of pDCs and IFNs during chronic HIV/SIV infection is also well appreciated, where persistent IFN production and ISG expression correlates with higher viral load, hyperimmune activation, decreased CD4+ T cell counts, dysregulated thymopoiesis, and disease progression (239, 259–262).

On a cellular level, HIV has been suggested to retain pDCs in an immature state that chronically produces IFN-Is (263–265), as part of a working model where pDCs become differentially activated into IFN-producing or antigen-presenting cells based on the subcellular compartment pathogens are sensed in (266). Endosomal sensing, such as for HIV, triggers IRF7 signaling and IFN induction leading to retention in an immature “non-DC-like” state whilst engagement in the lysosomes leads to NF-κB-mediated transcription of pro-inflammatory cytokines and maturation into a professional antigen presenting cell (265, 266). How this corresponds with previous reports of (1) HIV transfer to CD4^+^ T cells, (2) HIV antigen presentation to CD4^+^ T cells, and (3) HIV-induced increases in CD80, CD83, and CD86 expression by pDCs (247, 248, 267–270) was initially unclear, but may be explained by our new understanding of pDC subsets (P1-P3) and separation from Axl^+^ DCs. P1 pDCs may represent the majority of the IFN-producing cells during HIV, responsible for the apparent protective but multifaceted effects of early IFN-I during infection. P3 pDCs and Axl^+^ DCs may account for the cDC-like functions of pDCs during infection, namely viral transfer and cross-presentation and production of pro-inflammatory cytokines—upon TLR7 stimulation, Axl^+^ DCs are able to produce IL-12 but not pDCs, and it is unclear whether P3 activated pDCs are also capable of this function alongside their potential for T cell stimulation. P2 pDCs may encompass the effects of both P1 and P3 pDCs as a functional intermediate. It is worth noting all three pDC subsets are also able to produce TNF-α following viral stimulation (60), and thus are all likely to play a role in TNF-mediated inflammation during HIV infection.

It is tempting to speculate about the early involvement Axl^+^ DCs may have during HIV infection, given they appear to have similar migratory patterns to pDCs (absent in healthy skin but present in lymphoid tissue) (17). CD11c^hi^ Axl+ DCs have a cDC-like gene signature (14), which suggests they may play a similar role to other myeloid DCs in infection, namely efficiently transferring virus to CD4^+^ T cells and disseminating infection to other physical compartments such as the lymph nodes. In contrast, CD123^hi^ Axl^+^ DCs have a gene signature that aligns more closely with pDCs (14)—given they do not produce IFNs in response to HIV, whether they resemble other pDC functions during infection and still possess the potential for viral transfer would be interesting to determine. Whether Axl^+^ DCs experience productive HIV infection (and hence can undergo second-phase transfer) like other myeloid DCs is also unknown. The characterisation of the cytokines and chemokines produced by P1-P3 pDCs and Axl^+^ DCs in response to HIV and their susceptibility to infection will be critical for unraveling the role of each of these cell types during early and later stages of HIV infection.

## Concluding Remarks

Dendritic cells have most commonly been recognized for their ability to stimulate antigen-specific T cell responses, thereby forming a strong link between innate and adaptive immunity. Like the complexity of this role and other immunomodulatory effects they exert, the repertoire of DCs across different compartments has been difficult to comprehensively capture. However, building on countless years of previous work, we have seen great advances in our conceptualization of the DC spectrum through the emergence of powerful single-cell technologies (one of the most prominent being single-cell RNA sequencing). In peripheral blood, what originally comprised of 3 key DC subsets (CD141^+^ cDC1, CD1c^+^ cDC2, and CD123^+^ pDC) has now been expanded to 6 putative subsets (cDC1, cDC2-A/B, CD16^+^ DC, Axl^+^ DC, and pDC) which can be distinguished by expression of CD11c, CD16, Clec9a/CADM1, CD1c, CD32b, CD163, Axl, Siglec6, and CD123. Heterogeneity of blood DCs also extends to their developmental relationships, which will require further validation to accurately trace the precursor identities of each fully-differentiated DC. In peripheral tissue, each tissue site contains a discrete collection of DCs, ranging from Langerhans cells and other DC subsets in the outer epidermis of skin and Type II mucosa, to dermal and *lamina propria* cDC1s, cDC2s, and CD14^+^ cells, as well as their intestinal counterparts defined by CD103 and SIRPα expression.

For a comprehensive understanding of their roles in immunity, it is imperative that we begin to match and dissect these new DC subsets to previous descriptions of their immune functions particularly during disease and infection. In the context of HIV, the relative contributions of Langerhans cells and other epidermal DCs (such as VEDCs) to HIV transmission at the earliest stages must be re-examined, and similarly with pDCs and Axl^+^ DCs. Indeed, what are each of their roles in (1) HIV transfer to CD4^+^ T cells, (2) the secretion of pro-inflammatory and antiviral cytokines, and (3) the recruitment of CD4^+^ T cells and other HIV targets at the site of infection. By continuously updating our view of DC subsets and development, we can better understand how they influence infection with HIV and other pathogens, and thus precisely modulate their behavior to protect us from disease.

## Author Contributions

JR and OT primarily wrote the article under the guidance of AH and ST.

### Conflict of Interest Statement

The authors declare that the research was conducted in the absence of any commercial or financial relationships that could be construed as a potential conflict of interest.

## References

[1] Langerhans P Uber die nerven der menschlichen haut. Arch Pathol Anatomy. (1868) 44:325–7. 10.1007/BF01959006

[2] Steinman R Cohn Z. Identification of a novel cell type in peripheral lymphoid organs of mice. I. Morphology, quantitation, tissue distribution. J Exp Med. (1973) 137:1142–62. 10.1084/jem.137.5.1142 4573839 PMC2139237

[3] Steinman R Cohn Z. Identification of a novel cell type in peripheral lymphoid organs of mice. II. Functional properties *in vitro*. J Exp Med. (1974) 139:380–97. 10.1084/jem.139.2.380 4589990 PMC2139525

[4] Hart D Fabre J Demonstration and characterization of Ia-positive dendritic cells in the interstitial connective tissues of rat heart and other tissues, but not brain. J Exp Med. (1981) 154:347–61. 10.1084/jem.154.2.347 6943285 PMC2186417

[5] Szakal A Hanna M. The ultrastructure of antigen localization and viruslike particles in mouse spleen germinal centers. Exp Mol Pathol. (1968) 8:75–89. 10.1016/0014-4800(68)90007-5 4170142

[6] Nossal G Abbot A Mitchell J Lummus Z. Antigens in immunity. XV. Ultrastructural features of antigen capture in primary and secondary lymphoid follicles. J Exp Med. (1968) 127:277–90. 10.1084/jem.127.2.277 4169585 PMC2138444

[7] Lennert K Remmele W: karyometrische untersuchungen an lymphknotenzellen des menschen Acta Haematol. (1958) 19:99–113. 10.1159/000205419 13520253

[8] O'Doherty U Peng M Gezelter S Swiggard W Betjes M Bhardwaj N. Human blood contains two subsets of dendritic cells, one immunologically mature and the other immature. Immunology. (1994) 82:487–93. 7525461 PMC1414873

[9] Cella M Jarrossay D Facchetti F Alebardi O Nakajima H Lanzavecchia A. Plasmacytoid monocytes migrate to inflamed lymph nodes and produce large amounts of type I interferon. Nat Med. (1999) 5:919–23. 10.1038/11360 10426316

[10] Siegal F Kadowaki N Fitzgerald-Bocarsly P Shah K Ho S Antonenko S. The nature of the principal type 1 interferon-producing cells in human blood. Science. (1999) 284:1835–7. 10.1126/science.284.5421.1835 10364556

[11] Peters JH Ruhl S Friedrichs D. Veiled accessory cells deduced from monocytes. Immunobiology. (1987) 176:154–66. 10.1016/S0171-2985(87)80107-9 3502337

[12] Jaitin DA Kenigsberg E Keren-Shaul H Elefant N Paul F Zaretsky I. Massively parallel single-cell RNA-seq for marker-free decomposition of tissues into cell types. Science. (2014) 343:776–9. 10.1126/science.1247651 24531970 PMC4412462

[13] Zheng GX Terry JM Belgrader P Ryvkin P Bent ZW Wilson R. Massively parallel digital transcriptional profiling of single cells. Nat Commun. (2017) 8:14049. 10.1038/ncomms14049 28091601 PMC5241818

[14] Villani A-CC Satija R Reynolds G Sarkizova S Shekhar K Fletcher J. Single-cell RNA-seq reveals new types of human blood dendritic cells, monocytes, and progenitors. Science. (2017) 356:4573. 10.1126/science.aah4573 28428369 PMC5775029

[15] Björklund ÅK Forkel M Picelli S Konya V Theorell J Friberg D. The heterogeneity of human CD127(+) innate lymphoid cells revealed by single-cell RNA sequencing. Nat Immunol. (2016) 17:451–60. 10.1038/ni.3368 26878113

[16] Crinier A Milpied P Escalière B Piperoglou C Galluso J Balsamo A. High-dimensional single-cell analysis identifies organ-specific signatures and conserved NK cell subsets in humans and mice. Immunity. (2018) 49:971–86.e5. 10.1016/j.immuni.2018.09.009 30413361 PMC6269138

[17] Alcántara-Hernández M Leylek R Wagar LE Engleman EG Keler T Marinkovich M. High-dimensional phenotypic mapping of human dendritic cells reveals interindividual variation and tissue specialization. Immunity. (2017) 47:1037–50.e6. 10.1016/j.immuni.2017.11.001 29221729 PMC5738280

[18] Gury-BenAri M Thaiss CA Serafini N Winter DR Giladi A Lara-Astiaso D. The spectrum and regulatory landscape of intestinal innate lymphoid cells are shaped by the microbiome. Cell. (2016) 166:1231–46.e13. 10.1016/j.cell.2016.07.043 27545347

[19] Keren-Shaul H Spinrad A Weiner A Matcovitch-Natan O Dvir-Szternfeld R Ulland TK. A Unique microglia type associated with restricting development of alzheimer's disease. Cell. (2017) 169:1276–90.e17. 10.1016/j.cell.2017.05.018 28602351

[20] Gaublomme JT Yosef N Lee Y Gertner RS Yang LV Wu C. Single-cell genomics unveils critical regulators of Th17 cell pathogenicity. Cell. (2015) 163:1400–12. 10.1016/j.cell.2015.11.009 26607794 PMC4671824

[21] Giladi A Paul F Herzog Y Lubling Y Weiner A Yofe I. Single-cell characterization of haematopoietic progenitors and their trajectories in homeostasis and perturbed haematopoiesis. Nat Cell Biol. (2018) 20:836–46. 10.1038/s41556-018-0121-4 29915358

[22] Stubbington MJ Lönnberg T Proserpio V Clare S Speak AO Dougan G. T cell fate and clonality inference from single-cell transcriptomes. Nat Methods. (2016) 13:329–32. 10.1038/nmeth.3800 26950746 PMC4835021

[23] Herman JS Sagar Grün D. FateID infers cell fate bias in multipotent progenitors from single-cell RNA-seq data. Nat Methods. (2018) 15:379–86. 10.1038/nmeth.4662 29630061

[24] Rodrigues PF Alberti-Servera L Eremin A Grajales-Reyes GE Ivanek R Tussiwand R. Distinct progenitor lineages contribute to the heterogeneity of plasmacytoid dendritic cells. Nat Immunol. (2018) 19:711–22. 10.1038/s41590-018-0136-9 29925996 PMC7614340

[25] Haniffa M Collin M Ginhoux F. Ontogeny and functional specialization of dendritic cells in human and mouse. Adv Immunol. (2013) 120:1–49. 10.1016/B978-0-12-417028-5.00001-6 24070379

[26] Tussiwand R Gautier EL. Transcriptional regulation of mononuclear phagocyte development. Front Immunol. (2015) 6:533. 10.3389/fimmu.2015.00533 26539196 PMC4609886

[27] Murphy TL Grajales-Reyes GE Wu X Tussiwand R Briseño CG Iwata A. Transcriptional control of dendritic cell development. Ann Rev Immunol. (2016) 34:93–119. 10.1146/annurev-immunol-032713-120204 26735697 PMC5135011

[28] Swiecki M Colonna M. The multifaceted biology of plasmacytoid dendritic cells. Nat Rev Immunol. (2015) 15:471–85. 10.1038/nri3865 26160613 PMC4808588

[29] Bryant C Fromm PD Kupresanin F Clark G Lee K Clarke C. A CD2 high-expressing stress-resistant human plasmacytoid dendritic-cell subset. Immunol Cell Biol. (2016) 94:447–57. 10.1038/icb.2015.116 26791160

[30] Matsui T Connolly JE Michnevitz M Chaussabel D Yu C-II Glaser C. CD2 distinguishes two subsets of human plasmacytoid dendritic cells with distinct phenotype and functions. J Immunol. (2009) 182:6815–23. 10.4049/jimmunol.0802008 19454677 PMC2749454

[31] Yin X Yu H Jin X Li J Guo H Shi Q. Human blood CD1c+ dendritic cells encompass CD5high and CD5low subsets that differ significantly in phenotype, gene expression, and functions. J Immunol. (2017) 198:1553–1564. 10.4049/jimmunol.1600193 28087664

[32] Zhang X Lepelley A Azria E Lebon P Roguet G Schwartz O. Neonatal plasmacytoid dendritic cells (pDCs) display subset variation but can elicit potent anti-viral innate responses. PLoS ONE. (2013) 8:e52003. 10.1371/journal.pone.0052003 23326320 PMC3542339

[33] Zhang H Gregorio JD Iwahori T Zhang X Choi O Tolentino LL. A distinct subset of plasmacytoid dendritic cells induces activation and differentiation of B and T lymphocytes. Proc Natl Acad Sci USA. (2017) 114:1988–93. 10.1073/pnas.1610630114 28167780 PMC5338447

[34] MacDonald KP Munster DJ Clark GJ Dzionek A Schmitz J Hart DN. Characterization of human blood dendritic cell subsets. Blood. (2002) 100:4512–20. 10.1182/blood-2001-11-0097 12393628

[35] Sommer U Larsson B Tuve S Wehner R Zimmerman N Kramer M. Proinflammatory human 6-sulfo LacNAc-positive dendritic cells accumulate in intestinal acute graft-versus-host disease. Haematologica. (2014) 99:101071. 10.3324/haematol.2013.101071 24682513 PMC4040901

[36] Zoccali C Moissl U Chazot C Mallamaci F Tripepi G Arkossy O. Chronic fluid overload and mortality in ESRD. J Am Soc Nephrol. (2017) 28:2491–7. 10.1681/ASN.2016121341 28473637 PMC5533242

[37] Bachem A Güttler S Hartung E Ebstein F Schaefer M Tannert A. Superior antigen cross-presentation and XCR1 expression define human CD11c+CD141+ cells as homologues of mouse CD8+ dendritic cells. J Exp Med. (2010) 207:1273–81. 10.1084/jem.20100348 20479115 PMC2882837

[38] Poulin LF Salio M Griessinger E Anjos-Afonso F Craciun L Chen J-LL. Characterization of human DNGR-1+ BDCA3+ leukocytes as putative equivalents of mouse CD8alpha+ dendritic cells. J Exp Med. (2010) 207:1261–71. 10.1084/jem.20092618 20479117 PMC2882845

[39] Jongbloed SL Kassianos AJ Nald KJ Clark GJ Ju X Angel CE. Human CD141+ (BDCA-3)+ dendritic cells (DCs) represent a unique myeloid DC subset that cross-presents necrotic cell antigens. J Exp Med. (2010) 207:1247–60. 10.1084/jem.20092140 20479116 PMC2882828

[40] Ahrens S Zelenay S Sancho D Hanč P Kjær S Feest C. F-actin is an evolutionarily conserved damage-associated molecular pattern recognized by DNGR-1, a receptor for dead cells. Immunity. (2012) 36:635–45. 10.1016/j.immuni.2012.03.008 22483800

[41] Zhang J-GG Czabotar PE Policheni AN Caminschi I Wan SS Kitsoulis S. The dendritic cell receptor Clec9A binds damaged cells via exposed actin filaments. Immunity. (2012) 36:646–57. 10.1016/j.immuni.2012.03.009 22483802

[42] Hémont C Neel A Heslan M Braudeau C Josien R. Human blood mDC subsets exhibit distinct TLR repertoire and responsiveness. J Leukocyte Biol. (2013) 93:599–609. 10.1189/jlb.0912452 23341538

[43] Colletti NJ Liu H Gower AC Alekseyev YO Arendt CW Shaw MH. TLR3 signaling promotes the induction of unique human BDCA-3 dendritic cell populations. Front Immunol. (2016) 7:88. 10.3389/fimmu.2016.00088 27014268 PMC4789364

[44] Liu S Cai X Wu J Cong Q Chen X Li T. Phosphorylation of innate immune adaptor proteins MAVS, STING, and TRIF induces IRF3 activation. Science. (2015) 347:aaa2630. 10.1126/science.aaa2630 25636800

[45] Schrøder M Melum GR Landsverk OJ Bujko A Yaqub S Gran E. CD1c-expression by monocytes - implications for the use of commercial CD1c+ dendritic cell isolation kits. PLoS ONE. (2016) 11:e0157387. 10.1371/journal.pone.0157387 27311059 PMC4911075

[46] Worah K Mathan TS Manh TP Keerthikumar S Schreibelt G Tel J. Proteomics of human dendritic cell subsets reveals subset-specific surface markers and differential inflammasome function. Cell Rep. (2016) 16:2953–66. 10.1016/j.celrep.2016.08.023 27626665 PMC5039226

[47] Manh T-PP Elhmouzi-Younes J Urien C Ruscanu S Jouneau L Bourge M Defining mononuclear phagocyte subset homology across several distant warm-blooded vertebrates through comparative transcriptomics. Front Immunol. (2015) 6:299 10.3389/fimmu.2015.00299 26150816 PMC4473062

[48] Mahnke K Guo M Lee S Sepulveda H Swain S Nussenzweig M. The dendritic cell receptor for endocytosis, DEC-205, can recycle and enhance antigen presentation via major histocompatibility complex class II-positive lysosomal compartments. J Cell Biol. (2000) 151:673–84. 10.1083/jcb.151.3.673 11062267 PMC2185598

[49] Segura E Valladeau-Guilemond J Donnadieu M-HH Sastre-Garau X Soumelis V Amigorena S. Characterization of resident and migratory dendritic cells in human lymph nodes. J Exp Med. (2012) 209:653–60. 10.1084/jem.20111457 22430490 PMC3328358

[50] Harman AN Bye CR Nasr N Sandgren KJ Kim M Mercier SK. Identification of lineage relationships and novel markers of blood and skin human dendritic cells. J Immunol. (2013) 190:66–79. 10.4049/jimmunol.1200779 23183897

[51] Piccioli D Tavarini S Borgogni E Steri V Nuti S Sammicheli C. Functional specialization of human circulating CD16 and CD1c myeloid dendritic-cell subsets. Blood. (2007) 109:5371–9. 10.1182/blood-2006-08-038422 17332250

[52] Loughland JR Woodberry T Boyle MJ Tipping PE Piera KA Amante FH. Plasmodium falciparum activates CD16+ dendritic cells to produce tumor necrosis factor and interleukin-10 in subpatent malaria. J Infect Dis. (2019) 219:660–71. 10.1093/infdis/jiy555 30239833 PMC6339523

[53] Schäkel K von Kietzell M Hänsel A Ebling A Schulze L Haase M. Human 6-sulfo LacNAc-expressing dendritic cells are principal producers of early interleukin-12 and are controlled by erythrocytes. Immunity. (2006) 24:767–77. 10.1016/j.immuni.2006.03.020 16782032

[54] Döbel T Kunze A Babatz J Tränkner K Ludwig A Schmitz M. FcγRIII (CD16) equips immature 6-sulfo LacNAc-expressing dendritic cells (slanDCs) with a unique capacity to handle IgG-complexed antigens. Blood. (2013) 121:3609–18. 10.1182/blood-2012-08-447045 23460612

[55] Hofer TP Zawada AM Frankenberger M Skokann K Satzl AA Gesierich W. slan-defined subsets of CD16-positive monocytes: impact of granulomatous inflammation and M-CSF receptor mutation. Blood. (2015) 126:2601–10. 10.1182/blood-2015-06-651331 26443621

[56] van Leeuwen-Kerkhoff N Lundberg K Westers TM Kordasti S Bontkes HJ de Gruijl TD Transcriptional profiling reveals functional dichotomy between human slan+ non-classical monocytes and myeloid dendritic cells. J Leukocyte Biol. (2017) 102:1055–68. 10.1189/jlb.3MA0117-037R 28720687

[57] Calzetti F Tamassia N Micheletti A Finotti G Bianchetto-Aguilera F Cassatella MA. Human dendritic cell subset 4 (DC4) correlates to a subset of CD14dim/-CD16++ monocytes. J Allergy Clin Immunol. (2018) 141:2276–9.e3. 10.1016/j.jaci.2017.12.988 29366702

[58] Honda K Taniguchi T. IRFs: master regulators of signalling by Toll-like receptors and cytosolic pattern-recognition receptors. Nat Rev Immunol. (2006) 6:644–58. 10.1038/nri1900 16932750

[59] Gilliet M Cao W Liu Y-JJ. Plasmacytoid dendritic cells: sensing nucleic acids in viral infection and autoimmune diseases. Nat Rev Immunol. (2008) 8:594–606. 10.1038/nri2358 18641647

[60] Alculumbre SG Saint-André V Domizio J Vargas P Sirven P Bost P. Diversification of human plasmacytoid predendritic cells in response to a single stimulus. Nat Immunol. (2018) 19:63–75. 10.1038/s41590-017-0012-z 29203862

[61] Wimmers F Subedi N van Buuringen N Heister D Vivié J Beeren-Reinieren I. Single-cell analysis reveals that stochasticity and paracrine signaling control interferon-alpha production by plasmacytoid dendritic cells. Nat Commun. (2018) 9:3317. 10.1038/s41467-018-05784-3 30127440 PMC6102223

[62] Kim S Kaiser V Beier E Bechheim M Guenthner-Biller M Ablasser A. Self-priming determines high type I IFN production by plasmacytoid dendritic cells. Eur J Immunol. (2014) 44:807–18. 10.1002/eji.201343806 24338737 PMC4523003

[63] Chao M Seita J Weissman I. Establishment of a normal hematopoietic and leukemia stem cell hierarchy. Cold Spring Harbor Sympos Quant Biol. (2008) 73:439–49. 10.1101/sqb.2008.73.031 19022770

[64] Morrison S Uchida N Weissman I. The biology of hematopoietic stem cells. Ann Rev Cell Dev Biol. (1995) 11:35–71. 10.1146/annurev.cb.11.110195.000343 8689561

[65] Kondo M Weissman I Akashi K. Identification of clonogenic common lymphoid progenitors in mouse bone marrow. Cell. (1997) 91:661–72. 10.1016/S0092-8674(00)80453-5 9393859

[66] Akashi K Traver D Miyamoto T Weissman I. A clonogenic common myeloid progenitor that gives rise to all myeloid lineages. Nature. (2000) 404:193–7. 10.1038/35004599 10724173

[67] Doulatov S Notta F Eppert K Nguyen LT Ohashi PS Dick JE. Revised map of the human progenitor hierarchy shows the origin of macrophages and dendritic cells in early lymphoid development. Nat Immunol. (2010) 11:585–93. 10.1038/ni.1889 20543838

[68] Notta F Doulatov S Laurenti E Poeppl A Jurisica I Dick JE. Isolation of single human hematopoietic stem cells capable of long-term multilineage engraftment. Science. (2011) 333:218–21. 10.1126/science.1201219 21737740

[69] Meredith MM Liu K Darrasse-Jeze G Kamphorst AO Schreiber HA Guermonprez P. Expression of the zinc finger transcription factor zDC (Zbtb46, Btbd4) defines the classical dendritic cell lineage. J Exp Med. (2012) 209:1153–65. 10.1084/jem.20112675 22615130 PMC3371731

[70] Satpathy AT Wumesh K Albring JC Edelson BT Kretzer NM Bhattacharya D. Zbtb46 expression distinguishes classical dendritic cells and their committed progenitors from other immune lineages. J Exp Med. (2012) 209:1135–52. 10.1084/jem.20120030 22615127 PMC3371733

[71] Cisse B Caton ML Lehner M Maeda T Scheu S Locksley R. Transcription factor E2-2 is an essential and specific regulator of plasmacytoid dendritic cell development. Cell. (2008) 135:37–48. 10.1016/j.cell.2008.09.016 18854153 PMC2631034

[72] Hacker C Kirsch RD Ju X-SS Hieronymus T Gust TC Kuhl C. Transcriptional profiling identifies Id2 function in dendritic cell development. Nat Immunol. (2003) 4:380–6. 10.1038/ni903 12598895

[73] Hildner K Edelson BT Purtha WE Diamond M Matsushita H Kohyama M. Batf3 deficiency reveals a critical role for CD8alpha+ dendritic cells in cytotoxic T cell immunity. Science. (2008) 322:1097–100. 10.1126/science.1164206 19008445 PMC2756611

[74] Schlitzer A McGovern N Teo P Zelante T Atarashi K Low D. IRF4 transcription factor-dependent CD11b+ dendritic cells in human and mouse control mucosal IL-17 cytokine responses. Immunity. (2013) 38:970–83. 10.1016/j.immuni.2013.04.011 23706669 PMC3666057

[75] Sichien D Scott CL Martens L Vanderkerken M Gassen S Plantinga M. IRF8 transcription factor controls survival and function of terminally differentiated conventional and plasmacytoid dendritic cells, respectively. Immunity. (2016) 45:626–40. 10.1016/j.immuni.2016.08.013 27637148

[76] Tussiwand R Everts B Grajales-Reyes GE Kretzer NM Iwata A Bagaitkar J. Klf4 expression in conventional dendritic cells is required for T helper 2 cell responses. Immunity. (2015) 42:916–28. 10.1016/j.immuni.2015.04.017 25992862 PMC4447135

[77] Sallusto F Lanzavecchia A. Efficient presentation of soluble antigen by cultured human dendritic cells is maintained by granulocyte/macrophage colony-stimulating factor plus interleukin 4 and downregulated by tumor necrosis factor alpha. J Exp Med. (1994) 179:1109–18. 10.1084/jem.179.4.1109 8145033 PMC2191432

[78] Menezes S Melandri D Anselmi G Perchet T Loschko J Dubrot J The heterogeneity of Ly6Chi monocytes controls their differentiation into iNOS+ macrophages or monocyte-derived dendritic cells. Immunity. (2016) 45:1205–18. 10.1016/j.immuni.2016.12.001 28002729 PMC5196026

[79] Singh TP Zhang HH Borek I Wolf P Hedrick MN Singh SP. Monocyte-derived inflammatory Langerhans cells and dermal dendritic cells mediate psoriasis-like inflammation. Nat Commun. (2016) 7:13581. 10.1038/ncomms13581 27982014 PMC5171657

[80] Wacleche VS Cattin A Goulet J-PP Gauchat D Gosselin A Cleret-Buhot A. CD16+ monocytes give rise to CD103+RALDH2+TCF4+ dendritic cells with unique transcriptional and immunological features. Blood Adv. (2018) 2:2862–78. 10.1182/bloodadvances.2018020123 30381402 PMC6234376

[81] Bassler K Schulte-Schrepping J Warnat-Herresthal S Aschenbrenner AC Schultze JL The myeloid cell compartment-cell by cell. Ann Rev Immunol. (2019) 2019:41728 10.1146/annurev-immunol-042718-041728 30649988

[82] Naik SH Sathe P Park H-YY Metcalf D Proietto AI Dakic A. Development of plasmacytoid and conventional dendritic cell subtypes from single precursor cells derived *in vitro* and *in vivo*. Nat Immunol. (2007) 8:1217–26. 10.1038/ni1522 17922015

[83] Onai N Obata-Onai A Schmid MA Ohteki T Jarrossay D Manz MG. Identification of clonogenic common Flt3+M-CSFR+ plasmacytoid and conventional dendritic cell progenitors in mouse bone marrow. Nat Immunol. (2007) 8:1207–16. 10.1038/ni1518 17922016

[84] Onai N Kurabayashi K Hosoi-Amaike M Toyama-Sorimachi N Matsushima K Inaba K. A clonogenic progenitor with prominent plasmacytoid dendritic cell developmental potential. Immunity. (2013) 38:943–57. 10.1016/j.immuni.2013.04.006 23623382

[85] Schlitzer A Loschko J Mair K Vogelmann R Henkel L Einwächter H. Identification of CCR9- murine plasmacytoid DC precursors with plasticity to differentiate into conventional DCs. Blood. (2011) 117:6562–70. 10.1182/blood-2010-12-326678 21508410

[86] Loschko J Rieke GJ Schreiber HA Meredith MM Yao K-HH Guermonprez P. Inducible targeting of cDCs and their subsets *in vivo*. J Immunol Methods. (2016) 434:32–8. 10.1016/j.jim.2016.04.004 27073171 PMC4902770

[87] Sawai C Babovic S Upadhaya S Knapp DJ Lavin Y Lau CM. Hematopoietic stem cells are the major source of multilineage hematopoiesis in adult animals. Immunity. (2016) 45:597–609. 10.1016/j.immuni.2016.08.007 27590115 PMC5054720

[88] Upadhaya S Wai C Papalexi E Rashidfarrokhi A Jang G Chattopadhyay P. Kinetics of adult hematopoietic stem cell differentiation *in vivo*. J Exp Med. (2018) 215:2815–32. 10.1084/jem.20180136 30291161 PMC6219744

[89] Salvermoser J van Blijswijk J Papaioannou NE Rambichler S Pasztoi M Pakalniškyte D. Clec9a-mediated ablation of conventional dendritic cells suggests a lymphoid path to generating dendritic cells *in vivo*. Front Immunol. (2018) 9:699. 10.3389/fimmu.2018.00699 29713321 PMC5911463

[90] Helft J Anjos-Afonso F van der Veen Chakravarty P Bonnet D e Sousa C. Dendritic cell lineage potential in human early hematopoietic progenitors. Cell Rep. (2017) 20:529–37. 10.1016/j.celrep.2017.06.075 28723558 PMC5529209

[91] Dekker JD Rhee C Hu Z Lee B-K Lee J Iyer VR Lymphoid origin of a lineage of intrinsically activated plasmacytoid dendritic cell in mice and humans. bioRxiv. (2018) 2018:310680 10.1101/310680 PMC11398865 39269281

[92] Sathe P Metcalf D Vremec D Naik SH Langdon WY Huntington ND Lymphoid tissue and plasmacytoid dendritic cells and macrophages do not share a common macrophage-dendritic cell-restricted progenitor. Immunity. (2014) 41:104–15. 10.1016/j.immuni.2014.05.020 25035955

[93] Lee J Zhou YJ Ma W Zhang W Aljoufi A Luh T Lineage specification of human dendritic cells is marked by IRF8 expression in hematopoietic stem cells and multipotent progenitors. Nat Immunol. (2017) 18:877–88. 10.1038/ni.3789 28650480 PMC5743223

[94] Paul F Arkin Y Giladi A Jaitin DA Kenigsberg E Keren-Shaul H. Transcriptional heterogeneity and lineage commitment in myeloid progenitors. Cell. (2015) 163:1663–77. 10.1016/j.cell.2015.11.013 26627738

[95] Notta F Zandi S Takayama N Dobson S Gan OI Wilson G. Distinct routes of lineage development reshape the human blood hierarchy across ontogeny. Science. (2016) 351:aab2116. 10.1126/science.aab2116 26541609 PMC4816201

[96] Velten L Haas SF Raffel S Blaszkiewicz S Islam S Hennig BP. Human haematopoietic stem cell lineage commitment is a continuous process. Nat Cell Biol. (2017) 19:271–81. 10.1038/ncb3493 28319093 PMC5496982

[97] Karamitros D Stoilova B Aboukhalil Z Hamey F Reinisch A Samitsch M. Single-cell analysis reveals the continuum of human lympho-myeloid progenitor cells. Nat Immunol. (2018) 19:85–97. 10.1038/s41590-017-0001-2 29167569 PMC5884424

[98] Lin DS Kan A Gao J Crampin EJ Hodgkin PD Naik SH. DiSNE movie visualization and assessment of clonal kinetics reveal multiple trajectories of dendritic cell development. Cell Rep. (2018) 22:2557–66. 10.1016/j.celrep.2018.02.046 29514085

[99] Naik SH Perié L Swart E Gerlach C van Rooij N de Boer RJ. Diverse and heritable lineage imprinting of early haematopoietic progenitors. Nature. (2013) 496:229–32. 10.1038/nature12013 23552896

[100] Artyomov MN Munk A Gorvel L Korenfeld D Cella M Tung T. Modular expression analysis reveals functional conservation between human Langerhans cells and mouse cross-priming dendritic cells. J Exp Med. (2015) 212:743–57. 10.1084/jem.20131675 25918340 PMC4419344

[101] Carpentier S Manh T-P Chelbi R Henri S Malissen B Haniffa M. Comparative genomics analysis of mononuclear phagocyte subsets confirms homology between lymphoid tissue-resident and dermal XCR1+ DCs in mouse and human and distinguishes them from Langerhans cells. J Immunol Methods. (2016) 432:35–49. 10.1016/j.jim.2016.02.023 26966045 PMC4859332

[102] Chu C-C Ali N Karagiannis P Meglio P Skowera A Napolitano L. Resident CD141 (BDCA3)+ dendritic cells in human skin produce IL-10 and induce regulatory T cells that suppress skin inflammation. J Exp Med. (2012) 209:935–45. 10.1084/jem.20112583 22547651 PMC3348099

[103] Haniffa M Shin A Bigley V McGovern N Teo P Immunity SP. Human tissues contain CD141hi cross-presenting dendritic cells with functional homology to mouse CD103+ nonlymphoid dendritic cells. Immunity. (2012) 37:60–73. 10.1016/j.immuni.2012.04.012 22795876 PMC3476529

[104] Botting RA Bertram KM Baharlou H Sandgren KJ Fletcher J Rhodes JW. Phenotypic and functional consequences of different isolation protocols on skin mononuclear phagocytes. J Leukocyte Biol. (2017) 101:1393–403. 10.1189/jlb.4A1116-496R 28270408 PMC5433859

[105] Mass E Ballesteros I Farlik M Halbritter F Günther P Crozet L. Specification of tissue-resident macrophages during organogenesis. Science. (2016) 353:aaf4238. 10.1126/science.aaf4238 27492475 PMC5066309

[106] Guilliams M Dutertre C-A Scott CL McGovern N Sichien D Chakarov S. Unsupervised high-dimensional analysis aligns dendritic cells across tissues and species. Immunity. (2016) 45:669–84. 10.1016/j.immuni.2016.08.015 27637149 PMC5040826

[107] Schulz C Perdiguero E Chorro L Szabo-Rogers H Cagnard N Kierdorf K. A lineage of myeloid cells independent of Myb and hematopoietic stem cells. Science. (2012) 336:86–90. 10.1126/science.1219179 22442384

[108] Geissmann F Prost C Monnet J-P Dy M Brousse N Hermine O. Transforming growth factor β1, in the presence of granulocyte/macrophage colony-stimulating factor and interleukin 4, induces differentiation of human peripheral blood monocytes into dendritic langerhans cells. J Exp Med. (1998) 187:961–6. 10.1084/jem.187.6.961 9500798 PMC2212193

[109] Martínez-Cingolani C Grandclaudon M Jeanmougin M Jouve M Zollinger R Soumelis V. Human blood BDCA-1 dendritic cells differentiate into Langerhans-like cells with thymic stromal lymphopoietin and TGF-β. Blood. (2014) 124:2411–20. 10.1182/blood-2014-04-568311 25114264

[110] Milne P Bigley V Gunawan M Haniffa M Blood CM. CD1c+ blood dendritic cells have Langerhans cell potential. Blood. (2015) 125:470–3. 10.1182/blood-2014-08-593582 25352125 PMC4358967

[111] Bigley V McGovern N Milne P Dickinson R Pagan S Cookson S. Langerin-expressing dendritic cells in human tissues are related to CD1c+ dendritic cells and distinct from Langerhans cells and CD141high XCR1+ dendritic cells. J Leukocyte Biol. (2015) 97:627–34. 10.1189/jlb.1HI0714-351R 25516751 PMC4370053

[112] Flacher V Bouschbacher M Verronèse E Massacrier C Sisirak V Berthier-Vergnes O. Human Langerhans cells express a specific TLR profile and differentially respond to viruses and Gram-positive bacteria. J Immunol. (2006) 177:7959–67. 10.4049/jimmunol.177.11.7959 17114468

[113] Ouchi T Kubo A Yokouchi M. Langerhans cell antigen capture through tight junctions confers preemptive immunity in experimental staphylococcal scalded skin syndrome. J Exp Med. (2011) 208:2607–13. 10.1084/jem.20111718 22143886 PMC3244045

[114] Kubo A Nagao K Yokouchi M. External antigen uptake by Langerhans cells with reorganization of epidermal tight junction barriers. J Exp Med. (2009) 206:2937–46. 10.1084/jem.20091527 19995951 PMC2806471

[115] Fujita H Nograles KE Kikuchi T Gonzalez J Carucci JA Krueger JG. Human Langerhans cells induce distinct IL-22-producing CD4+ T cells lacking IL-17 production. Proc Natl Acad Sci USA. (2009) 106:21795–800. 10.1073/pnas.0911472106 19996179 PMC2799849

[116] Furio L Briotet I Journeaux A Billard H Péguet-Navarro J. Human langerhans cells are more efficient than CD14(-)CD1c(+) dermal dendritic cells at priming naive CD4(+) T cells. J Invest Dermatol. (2010) 130:1345–54. 10.1038/jid.2009.424 20107482

[117] Morelli AE Rubin J Erdos G Tkacheva OA Mathers AR Zahorchak AF. CD4+ T cell responses elicited by different subsets of human skin migratory dendritic cells. J Immunol. (2005) 175:7905–15. 10.4049/jimmunol.175.12.7905 16339526

[118] Mathers AR Janelsins BM Rubin JP Tkacheva OA Shufesky WJ Watkins SC. Differential capability of human cutaneous dendritic cell subsets to initiate Th17 responses. J Immunol. (2009) 182:921–33. 10.4049/jimmunol.182.2.921 19124735

[119] Kautz-Neu K Noordegraaf M. Langerhans cells are negative regulators of the anti-Leishmania response. J Exp Med. (2011) 208:885–91. 10.1084/jem.20102318 21536741 PMC3092359

[120] Price J Idoyaga J Salmon H Nature HB. CDKN1A regulates Langerhans cell survival and promotes Treg cell generation upon exposure to ionizing irradiation. Nat Immunol. (2015) 16:1060–8. 10.1038/ni.3270 26343536 PMC4620552

[121] Pena-Cruz V Agosto LM Akiyama H Olson A Moreau Y Larrieux J-R. HIV-1 replicates and persists in vaginal epithelial dendritic cells. J Clin Invest. (2018) 128:3439–44. 10.1172/JCI98943 29723162 PMC6063466

[122] Thépaut M Valladeau J Nurisso A Kahn R Arnou B Vivès C. Structural studies of langerin and Birbeck granule: a macromolecular organization model. Biochemistry. (2009) 48:2684–98. 10.1021/bi802151w 19175323

[123] Valladeau J Dezutter-Dambuyant C Saeland S. Langerin/CD207 sheds light on formation of birbeck granules and their possible function in Langerhans cells. Immunol Res. (2003) 28:93–107. 10.1385/IR:28:2:93 14610287

[124] Wollenberg A Kraft S Hanau D Bieber T. Immunomorphological and ultrastructural characterization of langerhans cells and a novel, inflammatory dendritic epidermal cell (IDEC) population in lesional skin of atopic eczema. J Invest Dermatol. (1996) 106:446–53. 10.1111/1523-1747.ep12343596 8648175

[125] Wollenberg A Oppel T Schottdorf E-M Günther S Moderer M Mommaas M. Expression and function of the mannose receptor CD206 on epidermal dendritic cells in inflammatory skin diseases. J Invest Dermatol. (2002) 118:327–34. 10.1046/j.0022-202x.2001.01665.x 11841552

[126] Yoshida K Kubo A Fujita H Yokouchi M Ishii K Kawasaki H. Distinct behavior of human Langerhans cells and inflammatory dendritic epidermal cells at tight junctions in patients with atopic dermatitis. J Allergy Clin Immunol. (2014) 134:856–64. 10.1016/j.jaci.2014.08.001 25282566

[127] Iwamoto K Nümm T Koch S Herrmann N Leib N Bieber T. Langerhans and inflammatory dendritic epidermal cells in atopic dermatitis are tolerized toward TLR2 activation. Allergy. (2018) 73:2205–13. 10.1111/all.13460 29672867

[128] Watchmaker PB Lahl K Lee M Baumjohann D Morton J Kim S. Comparative transcriptional and functional profiling defines conserved programs of intestinal DC differentiation in humans and mice. Nat Immunol. (2013) 15:ni.2768. 10.1038/ni.2768 24292363 PMC3942165

[129] Angel CE Lala A Chen C-JJ Edgar SG Ostrovsky LL Dunbar RP. CD14+ antigen-presenting cells in human dermis are less mature than their CD1a+ counterparts. Int Immunol. (2007) 19:1271–9. 10.1093/intimm/dxm096 17804688

[130] Turville SG Cameron PU Handley A Lin G Pöhlmann S Doms RW. Diversity of receptors binding HIV on dendritic cell subsets. Nat Immunol. (2002) 3:975–83. 10.1038/ni841 12352970

[131] Monte A Olivieri C-VV Vitale S Bailleux S Castillo L Giordanengo V. CD1c-related DCs that express CD207/langerin, but are distinguishable from langerhans cells, are consistently present in human tonsils. Front Immunol. (2016) 7:197. 10.3389/fimmu.2016.00197 27252701 PMC4879127

[132] Lundberg K Albrekt A-S Nelissen I Santegoets S de Gruijl TD Gibbs S. Transcriptional profiling of human dendritic cell populations and models - unique profiles of *in vitro* dendritic cells and implications on functionality and applicability. PLoS ONE. (2013) 8:e52875. 10.1371/journal.pone.0052875 23341914 PMC3544800

[133] Welty NE Staley C Ghilardi N Sadowsky MJ Igyártó BZ Kaplan DH Intestinal lamina propria dendritic cells maintain T cell homeostasis but do not affect commensalism. J Exp Med. (2013) 210:2011–24. 10.1084/jem.20130728 24019552 PMC3782055

[134] Haniffa M Ginhoux F Wang X-N Bigley V Abel M Dimmick I. Differential rates of replacement of human dermal dendritic cells and macrophages during hematopoietic stem cell transplantation. J Exp Med. (2009) 206:371–85. 10.1084/jem.20081633 19171766 PMC2646566

[135] McGovern N Schlitzer A Gunawan M Jardine L Shin A Poyner E. Human dermal CD14+ cells are a transient population of monocyte-derived macrophages. Immunity. (2014) 41:465–77. 10.1016/j.immuni.2014.08.006 25200712 PMC4175180

[136] Bujko A Atlasy N Landsverk O Richter L Yaqub S Horneland R. Transcriptional and functional profiling defines human small intestinal macrophage subsets. J Exp Med. (2017) 215:jem.20170057. 10.1084/jem.20170057 29273642 PMC5789404

[137] Richter L Landsverk O Atlasy N Bujko A Yaqub S Horneland R. Transcriptional profiling reveals monocyte-related macrophages phenotypically resembling DC in human intestine. Mucosal Immunol. (2018) 11:1512–23. 10.1038/s41385-018-0060-1 30038215

[138] Segura E Touzot M Bohineust A Cappuccio A Chiocchia G Hosmalin A. Human inflammatory dendritic cells induce Th17 cell differentiation. Immunity. (2013) 38:336–48. 10.1016/j.immuni.2012.10.018 23352235

[139] Serbina NV Salazar-Mather TP Biron CA Kuziel WA Pamer EG. TNF/iNOS-producing dendritic cells mediate innate immune defense against bacterial infection. Immunity. (2003) 19:59–70. 10.1016/S1074-7613(03)00171-7 12871639

[140] Lowes MA Chamian F Abello M Fuentes-Duculan J Lin S-L Nussbaum R. Increase in TNF-α and inducible nitric oxide synthase-expressing dendritic cells in psoriasis and reduction with efalizumab (anti-CD11a). Proc Natl Acad Sci USA. (2005) 102:19057–62. 10.1073/pnas.0509736102 16380428 PMC1323218

[141] Hänsel A Günther C Baran W Bidier M Lorenz H-M Schmitz M. Human 6-sulfo LacNAc (slan) dendritic cells have molecular and functional features of an important pro-inflammatory cell type in lupus erythematosus. J Autoimmun. (2013) 40:1–8. 10.1016/j.jaut.2012.07.005 22890025

[142] Hänsel A Günther C Ingwersen J Starke J Schmitz M Bachmann M. Human slan (6-sulfo LacNAc) dendritic cells are inflammatory dermal dendritic cells in psoriasis and drive strong Th17/Th1 T-cell responses. J Allergy Clin Immunol. (2011) 127:787–94.e9. 10.1016/j.jaci.2010.12.009 21377044

[143] Günther C Starke J Zimmermann N Schäkel K. Human 6-sulfo LacNAc (slan) dendritic cells are a major population of dermal dendritic cells in steady state and inflammation. Clin Exp Dermatol. (2012) 37:169–76. 10.1111/j.1365-2230.2011.04213.x 22188261

[144] Micheletti A Finotti G Calzetti F Lonardi S Zoratti E Bugatti M slan/M-DC8 + cells constitute a distinct subset of dendritic cells in human tonsils. Oncotarget. (2015) 7:161–75. 10.18632/oncotarget.6660 PMC4807990 26695549

[145] Geijtenbeek T Kwon D Torensma R van Vliet S van Duijnhoven G Middel J. DC-SIGN, a dendritic cell-specific HIV-1-binding protein that enhances trans-infection of T cells. Cell. (2000) 100:587–97. 10.1016/S0092-8674(00)80694-7 10721995

[146] Steinman R. DC-SIGN: a guide to some mysteries of dendritic cells. Cell. (2000) 100:491–4. 10.1016/S0092-8674(00)80684-4 10721985

[147] Kavanagh DG Bhardwaj N. A division of labor: DC subsets and HIV receptor diversity. Nat Immunol. (2002) 3:891–3. 10.1038/ni1002-891 12352961

[148] Tenner-Racz K Racz P Bofill M Schulz-Meyer A Kern P Weber J. HTLV-III/LAV viral antigens in lymph nodes of homosexual men with persistent generalized lymphadenopathy and AIDS. Am J Pathol. (1986) 123:9–15. 3008562 PMC1888153

[149] Biberfeld P Porwit-Ksiazek A Böttiger B Morfeldt-Månsson L Biberfeld G Immunohistopathology of lymph nodes in HTLV-III infected homosexuals with persistent adenopathy or AIDS. Cancer Res. (1985) 45:4665s−70s. 2410110

[150] Heesters BA Lindqvist M Vagefi PA Scully EP Schildberg FA Altfeld M. Follicular dendritic cells retain infectious HIV in cycling endosomes. PLoS Pathogens. (2015) 11:e1005285. 10.1371/journal.ppat.1005285 26623655 PMC4666623

[151] Tschachler E Groh V Popovic M Mann D Konrad K Safai B. Epidermal Langerhans cells–a target for HTLV-III/LAV infection. J Invest Dermatol. (1987) 88:233–7. 10.1111/1523-1747.ep12525402 3100656

[152] Schmitt D Dezutter-Dambuyant C Hanau D Schmitt DA Kolbe HV Kieny MF HIV envelope proteins are bound by human epidermal Langerhans cells by a binding site which different from the site on the CD4 molecule, and are internalized by receptor endocytosis. Compt Rendus L'Acad Sci. (1989) 308:269–75. 2543485

[153] Macatonia S Patterson S Knight S. Suppression of immune responses by dendritic cells infected with HIV. Immunology. (1989) 67:285–9. 2788124 PMC1385341

[154] Patterson S Gross J Bedford P Knight S. Morphology and phenotype of dendritic cells from peripheral blood and their productive and non-productive infection with human immunodeficiency virus type 1. Immunology. (1991) 72:361–7. 1709140 PMC1384396

[155] Macatonia S Patterson S Knight S. Primary proliferative and cytotoxic T-cell responses to HIV induced in vitro by human dendritic cells. Immunology. (1991) 74:399–406. 1769689 PMC1384631

[156] Cameron PU Freudenthal PS Barker JM Gezelter S Inaba K Steinman RM. Dendritic cells exposed to human immunodeficiency virus type-1 transmit a vigorous cytopathic infection to CD4+ T cells. Science. (1992) 257:1848. 10.1126/science.1352913 1352913

[157] Frank I Piatak M Stoessel H Romani N Bonnyay D Lifson J. Infectious and whole inactivated simian immunodeficiency viruses interact similarly with primate dendritic cells (DCs): differential intracellular fate of virions in mature and immature DCs. J Virol. (2002) 76:2936–51. 10.1128/JVI.76.6.2936-2951.2002 11861860 PMC135959

[158] Turville SG Santos JJ Frank I Cameron PU Wilkinson J Miranda-Saksena M. Immunodeficiency virus uptake, turnover, and 2-phase transfer in human dendritic cells. Blood. (2004) 103:2170–9. 10.1182/blood-2003-09-3129 14630806

[159] Mercier SK Donaghy H Botting RA Turville SG Harman AN Nasr N. The microvesicle component of HIV-1 inocula modulates dendritic cell infection and maturation and enhances adhesion to and activation of T lymphocytes. PLoS Pathogens. (2013) 9:e1003700. 10.1371/journal.ppat.1003700 24204260 PMC3798598

[160] Pope M Betjes M Romani N Hirmand H Cameron P Hoffman L. Conjugates of dendritic cells and memory T lymphocytes from skin facilitate productive infection with HIV-1. Cell. (1994) 78:389–98. 10.1016/0092-8674(94)90418-9 7914836

[161] Pope M Gezelter S Gallo N Hoffman L Einman R. Low levels of HIV-1 infection in cutaneous dendritic cells promote extensive viral replication upon binding to memory CD4+ T cells. J Exp Med. (1995) 182:2045–56. 10.1084/jem.182.6.2045 7500050 PMC2192232

[162] Reece J Handley A Anstee E Morrison W Crowe S Cameron P. HIV-1 selection by epidermal dendritic cells during transmission across human skin. J Exp Med. (1998) 187:1623–31. 10.1084/jem.187.10.1623 9584140 PMC2212292

[163] Kawamura T Cohen S Borris D Aquilino E Glushakova S Margolis L. Candidate microbicides block HIV-1 infection of human immature Langerhans cells within epithelial tissue explants. J Exp Med. (2000) 192:1491–500. 10.1084/jem.192.10.1491 11085750 PMC2193188

[164] Romani N Gruner S Brang D Kämpgen E Lenz A Trockenbacher B. Proliferating dendritic cell progenitors in human blood. J Exp Med. (1994) 180:83–93. 10.1084/jem.180.1.83 8006603 PMC2191538

[165] Blauvelt A Asada H Saville M Klaus-Kovtun V Altman D Yarchoan R. Productive infection of dendritic cells by HIV-1 and their ability to capture virus are mediated through separate pathways. J Clin Invest. (1997) 100:2043–53. 10.1172/JCI119737 9329969 PMC508395

[166] Curtis B Scharnowske S Watson A. Sequence and expression of a membrane-associated C-type lectin that exhibits CD4-independent binding of human immunodeficiency virus envelope glycoprotein gp120. Proc Natl Acad Sci USA. (1992) 89:8356–60. 10.1073/pnas.89.17.8356 1518869 PMC49917

[167] Turville S Arthos J Nald K Lynch G Naif H Clark G. HIV gp120 receptors on human dendritic cells. Blood. (2001) 98:2482–8. 10.1182/blood.V98.8.2482 11588046

[168] Izquierdo-Useros N Lorizate M Puertas MC Rodriguez-Plata MT Zangger N Erikson E. Siglec-1 is a novel dendritic cell receptor that mediates HIV-1 trans-infection through recognition of viral membrane gangliosides. PLoS Biol. (2012) 10:e1001448. 10.1371/journal.pbio.1001448 23271952 PMC3525531

[169] de Witte L Bobardt M Chatterji U Degeest G David G Geijtenbeek TB. Syndecan-3 is a dendritic cell-specific attachment receptor for HIV-1. Proc Natl Acad Sci USA. (2007) 104:19464–9. 10.1073/pnas.0703747104 18040049 PMC2148312

[170] Baldauf H-MM Pan X Erikson E Schmidt S Daddacha W Burggraf M. SAMHD1 restricts HIV-1 infection in resting CD4(+) T cells. Nat Med. (2012) 18:1682–7. 10.1038/nm.2964 22972397 PMC3828732

[171] Li D Schlaepfer E Audigé A Rochat M-AA Ivic S Knowlton CN. Vpx mediated degradation of SAMHD1 has only a very limited effect on lentiviral transduction rate in *ex vivo* cultured HSPCs. Stem Cell Res. (2015) 15:271–80. 10.1016/j.scr.2015.06.012 26207584 PMC4766840

[172] Harman AN Kraus M Bye CR Byth K Turville SG Tang O. HIV-1–infected dendritic cells show 2 phases of gene expression changes, with lysosomal enzyme activity decreased during the second phase. Blood. (2009) 114:85–94. 10.1182/blood-2008-12-194845 19436054 PMC2710958

[173] Nasr N Lai J Botting RA Mercier SK Harman AN Kim M. Inhibition of two temporal phases of HIV-1 transfer from primary langerhans cells to T cells: the role of langerin. J Immunol. (2014) 193:2554–64. 10.4049/jimmunol.1400630 25070850

[174] McDonald D Wu L Bohks SM KewalRamani VN Unutmaz D Hope TJ. Recruitment of HIV and its receptors to dendritic cell-T cell junctions. Science. (2003) 300:1295–7. 10.1126/science.1084238 12730499

[175] Martin N Welsch S Jolly C Briggs JA Vaux D Sattentau QJ. Virological synapse-mediated spread of human immunodeficiency virus type 1 between T cells is sensitive to entry inhibition. J Virol. (2010) 84:3516–27. 10.1128/JVI.02651-09 20089656 PMC2838118

[176] Burleigh L Lozach P-Y Schiffer C Staropoli I Pezo V Porrot F Infection of dendritic cells (DCs), not DC-SIGN-mediated internalization of human immunodeficiency virus, is required for long-term transfer of virus to T cells. J Virol. (2006) 80:2949–57. 10.1128/JVI.80.6.2949-2957.2006 16501104 PMC1395470

[177] Nobile C Petit C Moris A Skrabal K Abastado J-P Mammano F. Covert human immunodeficiency virus replication in dendritic cells and in DC-SIGN-expressing cells promotes long-term transmission to lymphocytes. J Virol. (2005) 79:5386–99. 10.1128/JVI.79.9.5386-5399.2005 15827153 PMC1082762

[178] Jolly C Mitar I Sattentau QJ. Adhesion molecule interactions facilitate human immunodeficiency virus type 1-induced virological synapse formation between T cells. J Virol. (2007) 81:13916–21. 10.1128/JVI.01585-07 17913807 PMC2168851

[179] Kwon DS Gregorio G Bitton N Hendrickson WA Littman DR. DC-SIGN-mediated internalization of HIV is required for trans-enhancement of T cell infection. Immunity. (2002) 16:135–44. 10.1016/S1074-7613(02)00259-5 11825572

[180] Cavrois M Neidleman J Kreisberg JF Greene WC. *In vitro* derived dendritic cells trans-infect CD4 T cells primarily with surface-bound HIV-1 virions. PLoS Pathogens. (2007) 3:e4. 10.1371/journal.ppat.0030004 17238285 PMC1779297

[181] Yu H Reuter MA Nald D. HIV Traffics through a specialized, surface-accessible intracellular compartment during trans-infection of T cells by mature dendritic cells. PLoS Pathogens. (2008) 4:e1000134. 10.1371/journal.ppat.1000134 18725936 PMC2515344

[182] de Witte L Nabatov A Pion M Fluitsma D de Jong MA de Gruijl T. Langerin is a natural barrier to HIV-1 transmission by Langerhans cells. Nat Med. (2007) 13:367–71. 10.1038/nm1541 17334373

[183] Liu R Paxton W Choe S Ceradini D Martin S Horuk R. Homozygous defect in HIV-1 coreceptor accounts for resistance of some multiply-exposed individuals to HIV-1 infection. Cell. (1996) 86:367–77. 10.1016/S0092-8674(00)80110-5 8756719

[184] Aggarwal A Iemma TL Shih I Newsome TP McAllery S Cunningham AL. Mobilization of HIV spread by diaphanous 2 dependent filopodia in infected dendritic cells. PLoS Pathogens. (2012) 8:e1002762. 10.1371/journal.ppat.1002762 22685410 PMC3369929

[185] Harman AN Kim M Nasr N Sandgren KJ Cameron PU. Tissue dendritic cells as portals for HIV entry. Rev Med Virol. (2013) 23:319–33. 10.1002/rmv.1753 23908074

[186] Keele BF Estes JD. Barriers to mucosal transmission of immunodeficiency viruses. Blood. (2011) 118:839–46. 10.1182/blood-2010-12-325860 21555745 PMC3148165

[187] Rinaldo CR. HIV-1 trans infection of CD4+ T cells by professional antigen presenting cells. Scientifica. (2013) 2013:1–30. 10.1155/2013/164203 24278768 PMC3820354

[188] Henry M Uthman A Ballaun C Stingl G Tschachler E. Epidermal langerhans cells of AIDS patients express HIV-1 regulatory and structural genes. J Invest Dermatol. (1994) 103:593–6. 10.1111/1523-1747.ep12396918 7930687

[189] Dezutter-Dambuyant C. *In vivo* and *in vitro* infection of human Langerhans cells by HIV-1. Adv Exp Med Biol. (1995) 378:447–51 10.1007/978-1-4615-1971-3_100 8526115

[190] Cimaerelli A Zambruno G Marconi A Girolomoni G Bertazzoni U Gianetti A Quantitation by competitive PCR of HIV-1 proviral DNA in epidermal Langerhans cells of HIV-infected patients. J Acquired Immune Deficiency Syndromes. (1994) 7:230–35. 8106964

[191] Berger R Gartner S Rappersberger K Foster CA Wolff K Stingl G. Isolation of human immunodeficiency virus type 1 from human epidermis: virus replication and transmission studies. J Invest Dermatol. (1992) 99:271–7. 10.1111/1523-1747.ep12616619 1512462

[192] Kawamura T Koyanagi Y Nakamura Y Ogawa Y Yamashita A Iwamoto T. Significant virus replication in langerhans cells following application of HIV to abraded skin: relevance to occupational transmission of HIV. J Immunol. (2008) 180:3297–304. 10.4049/jimmunol.180.5.3297 18292554

[193] Kawamura T Gulden FO Sugaya M McNamara DT Borris DL Lederman MM. R5 HIV productively infects Langerhans cells, and infection levels are regulated by compound CCR5 polymorphisms. Proc Natl Acad Sci. (2003) 100:8401–6. 10.1073/pnas.1432450100 12815099 PMC166241

[194] Peressin M Holl V Schmidt S Decoville T Mirisky D Lederle A. HIV-1 replication in langerhans and interstitial dendritic cells is inhibited by neutralizing and Fc-mediated inhibitory antibodies. J Virol. (2011) 85:1077–85. 10.1128/JVI.01619-10 21084491 PMC3020030

[195] Fischetti L Barry SM Hope TJ Shattock RJ. HIV-1 infection of human penile explant tissue and protection by candidate microbicides. AIDS. (2009) 23:319–28. 10.1097/QAD.0b013e328321b778 19114867 PMC4349942

[196] Patterson BK Landay A Siegel JN Flener Z Pessis D Chaviano A. Susceptibility to human immunodeficiency virus-1 infection of human foreskin and cervical tissue grown in explant culture. Am J Pathol. (2002) 161:867–73. 10.1016/S0002-9440(10)64247-2 12213715 PMC1867269

[197] Dinh MH Anderson MR McRaven MD Cianci GC McCoombe SG Kelley Z Visualization of HIV-1 interactions with penile and foreskin epithelia: clues for female-to-male HIV transmission. PLOS Pathogens. (2015) 2015:1004729 10.1371/journal.ppat.1004729 PMC4352059 25748093

[198] Zhou Z de Longchamps N Schmitt A Zerbib M Vacher-Lavenu M-C Bomsel M HIV-1 efficient entry in inner foreskin is mediated by elevated CCL5/RANTES that recruits T cells and fuels conjugate formation with langerhans cells. PLoS Pathogens. (2011) 2011:2100 10.1371/journal.ppat.1002100 PMC3128116 21738469

[199] Ganor Y Bomsel M. HIV-1 Transmission in the male genital tract. Am J Reproduc Immunol. (2011) 65:284–91. 10.1111/j.1600-0897.2010.00933.x 21114566

[200] Ganor Y Zhou Z Tudor D Schmitt A Vacher-Lavenu M-CC Gibault L Within 1 h, HIV-1 uses viral synapses to enter efficiently the inner, but not outer, foreskin mucosa and engages Langerhans-T cell conjugates. Mucosal Immunol. (2010) 3:506–22. 10.1038/mi.2010.32 20571487

[201] Ballweber L Robinson B Kreger A Fialkow M Lentz G McElrath JM. Vaginal langerhans cells nonproductively transporting HIV-1 mediate infection of T cells. J Virol. (2011) 85:13443–7. 10.1128/JVI.05615-11 21976645 PMC3233146

[202] Hladik F Sakchalathorn P Ballweber L Lentz G Fialkow M Eschenbach D. Initial events in establishing vaginal entry and infection by human immunodeficiency virus type-1. Immunity. (2007) 26:257–70. 10.1016/j.immuni.2007.01.007 17306567 PMC1885958

[203] Liu CM Prodger JL Tobian AA Abraham AG Kigozi G Hungate BA. Penile anaerobic dysbiosis as a risk factor for HIV infection. mBio. (2017) 8:17. 10.1128/mBio.00996-17 28743816 PMC5527312

[204] Cavarelli M Foglieni C Rescigno M Scarlatti G Cavarelli M Foglieni C R5 HIV-1 envelope attracts dendritic cells to cross the human intestinal epithelium and sample luminal virions via engagement of the CCR5. EMBO Mol Med. (2013) 2013:201202232 10.1002/emmm.201202232 PMC3662319 23606583

[205] Gurney KB Elliott J Nassanian H Song C Soilleux E McGowan I. Binding and transfer of human immunodeficiency virus by DC-SIGN+ cells in human rectal mucosa. J Virol. (2005) 79:5762–73. 10.1128/JVI.79.9.5762-5773.2005 15827191 PMC1082722

[206] Shen R Smythies LE Clements RH Novak L Smith PD Shen R. Dendritic cells transmit HIV-1 through human small intestinal mucosa. J Leukocyte Biol. (2010) 87:663–70. 10.1189/jlb.0909605 20007245 PMC2858307

[207] Shen R Kappes JC Smythies LE Richter HE Novak L Smith PD. Vaginal myeloid dendritic cells transmit founder HIV-1. J Virol. (2014) 88:14. 10.1128/JVI.00766-14 24741097 PMC4054437

[208] Shen R Richter HE Smith PD. Early HIV-1 target cells in human vaginal and ectocervical mucosa. Am J Reproduc Immunol. (2011) 65:261–7. 10.1111/j.1600-0897.2010.00939.x 21118402 PMC3077123

[209] Rodriguez-Garcia M Fortier JM Barr FD Wira CR Isolation of dendritic cells from the human female reproductive tract for phenotypical and functional studies. J Visual Exp. (2018) 13:133 10.3791/57100 PMC5931758 29608161

[210] Rodriguez-Garcia M Shen Z Barr F Boesch A Ackerman M Kappes J. Dendritic cells from the human female reproductive tract rapidly capture and respond to HIV. Mucos Immunol. (2017) 10:531–44. 10.1038/mi.2016.72 27579858 PMC5332537

[211] Trifonova RT Bollman B Barteneva NS Lieberman J Trifonova RT Bollman B. Myeloid cells in intact human cervical explants capture HIV and can transmit it to CD4 T cells. Front Immunol. (2018) 9:2719. 10.3389/fimmu.2018.02719 30532754 PMC6265349

[212] McKinnon LR Liebenberg LJ Yende-Zuma N Archary D Ngcapu S Sivro A Genital inflammation undermines the effectiveness of tenofovir gel in preventing HIV acquisition in women. Nat Med. (2018) 491:4506 10.1038/nm.4506 PMC5893390 29480895

[213] Passmore J-AS Jaspan HB Masson L Genital inflammation, immune activation and risk of sexual HIV acquisition. Curr Opin HIV AIDS. (2016) 232:156–62. 10.1097/COH.0000000000000232 PMC6194860 26628324

[214] Masson L Passmore J-AS Liebenberg LJ Werner L Baxter C Nold K Genital inflammation and the risk of HIV acquisition in women. Clin Infect Dis. (2015) 2015:260–9. 10.1093/cid/civ298 PMC4565995 25900168

[215] Sozzani S Vermi W Prete A Facchetti F. Trafficking properties of plasmacytoid dendritic cells in health and disease. Trends Immunol. (2010) 31:270–7. 10.1016/j.it.2010.05.004 20579936

[216] Seth S Oberdörfer L Hyde R Hoff K Thies V Worbs T. CCR7 essentially contributes to the homing of plasmacytoid dendritic cells to lymph nodes under steady-state as well as inflammatory conditions. J Immunol. (2011) 186:3364–72. 10.4049/jimmunol.1002598 21296980

[217] Umemoto E Otani K Ikeno T Garcia N Hayasaka H Bai Z. Constitutive plasmacytoid dendritic cell migration to the splenic white pulp is cooperatively regulated by CCR7- and CXCR4-mediated signaling. J Immunol. (2012) 189:191–9. 10.4049/jimmunol.1200802 22634622

[218] Diacovo TG Blasius AL Mak TW Cella M Colonna M. Adhesive mechanisms governing interferon-producing cell recruitment into lymph nodes. J Exp Med. (2005) 202:687–96. 10.1084/jem.20051035 16147979 PMC2212867

[219] Sisirak V Vey N Vanbervliet B Duhen T Puisieux I Homey B. CCR6/CCR10-mediated plasmacytoid dendritic cell recruitment to inflamed epithelia after instruction in lymphoid tissues. Blood. (2011) 118:5130–40. 10.1182/blood-2010-07-295626 21937703 PMC3217401

[220] Wendland M Czeloth N Mach N Malissen B Kremmer E Pabst O. CCR9 is a homing receptor for plasmacytoid dendritic cells to the small intestine. Proc Natl Acad Sci USA. (2007) 104:6347–52. 10.1073/pnas.0609180104 17404233 PMC1851094

[221] Krug A Uppaluri R Facchetti F Dorner BG Sheehan KC Schreiber RD. IFN-producing cells respond to CXCR3 ligands in the presence of CXCL12 and secrete inflammatory chemokines upon activation. J Immunol. (2002) 169:6079–83. 10.4049/jimmunol.169.11.6079 12444109

[222] Shang L Duan L Perkey K Wietgrefe S Zupancic M Smith A. Epithelium-innate immune cell axis in mucosal responses to SIV. Mucos Immunol. (2017) 10:508–19. 10.1038/mi.2016.62 27435105 PMC5250613

[223] Li Q Estes JD Hlievert P Duan L Brosnahan AJ Southern PJ. Glycerol monolaurate prevents mucosal SIV transmission. Nature. (2009) 458:1034–8. 10.1038/nature07831 19262509 PMC2785041

[224] Schmidt B Ashlock BM Foster H Fujimura SH Levy JA. HIV-infected cells are major inducers of plasmacytoid dendritic cell interferon production, maturation, and migration. Virology. (2005) 343:256–66. 10.1016/j.virol.2005.09.059 16278001

[225] Beignon AS McKenna Km Skoberne Manches O DaSilva I Kavanagh DG. Enocytosis of HIV-1 activates Plasmacytoid dendritic cells via Toll-like receptor-viral RNA interactions. J Clin Invest. (2005) 115:3265–75. 10.1172/JCI26032 16224540 PMC1253628

[226] Harper MS Guo K Gibbert K Lee EJ Llon S Barrett BS. Interferon-α subtypes in an *ex vivo* model of acute HIV-1 infection: expression, potency and effector mechanisms. PLoS Pathogens. (2015) 11:e1005254. 10.1371/journal.ppat.1005254 26529416 PMC4631339

[227] Boasso A Royle CM Doumazos S Aquino VN Biasin M Piacentini L. Overactivation of plasmacytoid dendritic cells inhibits antiviral T-cell responses: a model for HIV immunopathogenesis. Blood. (2011) 118:5152–62. 10.1182/blood-2011-03-344218 21931112 PMC3217402

[228] Gibson SJ Lindh JM Riter TR Gleason RM Rogers LM Fuller AE. Plasmacytoid dendritic cells produce cytokines and mature in response to the TLR7 agonists, imiquimod and resiquimod. Cell Immunol. (2002) 218:74–86. 10.1016/S0008-8749(02)00517-8 12470615

[229] Birmachu W Gleason RM Bulbulian BJ Riter CL Vasilakos JP Lipson KE. Transcriptional networks in plasmacytoid dendritic cells stimulated with synthetic TLR 7 agonists. BMC Immunol. (2007) 8:26. 10.1186/1471-2172-8-26 17935622 PMC2175514

[230] Doehle BP Hladik F McNevin JP McElrath M Gale M. Human immunodeficiency virus type 1 mediates global disruption of innate antiviral signaling and immune defenses within infected cells. J Virol. (2009) 83:10395–405. 10.1128/JVI.00849-09 19706707 PMC2753137

[231] Harman AN Nasr N Feetham A Galoyan A Alshehri AA Rambukwelle D. HIV blocks interferon induction in human dendritic cells and macrophages by dysregulation of TBK1. J Virol. (2015) 89:6575–84. 10.1128/JVI.00889-15 25855743 PMC4468486

[232] Doehle BP Chang K Rustagi A McNevin J McElrath M Gale M. Vpu mediates depletion of interferon regulatory factor 3 during HIV infection by a lysosome-dependent mechanism. J Virol. (2012) 86:8367–74. 10.1128/JVI.00423-12 22593165 PMC3421752

[233] Okumura A Alce T Lubyova B Ezelle H Strebel K Pitha PM. HIV-1 accessory proteins VPR and Vif modulate antiviral response by targeting IRF-3 for degradation. Virology. (2008) 373:85–97. 10.1016/j.virol.2007.10.042 18082865 PMC2312338

[234] Gringhuis SI Hertoghs N Kaptein TM Zijlstra-Willems EM Sarrami-Forooshani R Sprokholt JK HIV-1 blocks the signaling adaptor MAVS to evade antiviral host defense after sensing of abortive HIV-1 RNA by the host helicase DDX3. Nat Immunol. (2017) 18:225–35. 10.1038/ni.3647 28024153

[235] Lahaye X Satoh T Gentili M Cerboni S Conrad C Hurbain I. The capsids of HIV-1 and HIV-2 determine immune detection of the viral cDNA by the innate sensor cGAS in dendritic cells. Immunity. (2013) 39:1132–42. 10.1016/j.immuni.2013.11.002 24269171

[236] Harris LD Tabb B Sodora DL Paiardini M Klatt NR Douek DC. Downregulation of robust acute type I interferon responses distinguishes nonpathogenic simian immunodeficiency virus (SIV) infection of natural hosts from pathogenic SIV infection of rhesus macaques. J Virol. (2010) 84:7886–91. 10.1128/JVI.02612-09 20484518 PMC2897601

[237] Campillo-Gimenez L Laforge M Fay M Brussel A Cumont M-CC Monceaux V Nonpathogenesis of simian immunodeficiency virus infection is associated with reduced inflammation and recruitment of plasmacytoid dendritic cells to lymph nodes, not to lack of an interferon type I response, during the acute phase. J Virol. (2010) 84:1838–46. 10.1128/JVI.01496-09 19939930 PMC2812402

[238] Ferbas J Toso J Logar A Navratil J Rinaldo C. CD4+ blood dendritic cells are potent producers of IFN-alpha in response to in vitro HIV-1 infection. J Immunol. (1994) 152:4649–62. 7908920

[239] Sandler NG Bosinger SE Estes JD Zhu RT Tharp GK Boritz E. Type I interferon responses in rhesus macaques prevent SIV infection and slow disease progression. Nature. (2014) 511:601–5. 10.1038/nature13554 25043006 PMC4418221

[240] Carnathan D Lawson B Yu J Patel K Billingsley JM Tharp GK. Reduced chronic lymphocyte activation following interferon alpha blockade during the acute phase of simian immunodeficiency virus infection in rhesus macaques. J Virol. (2018) 92:17. 10.1128/JVI.01760-17 29467313 PMC5899190

[241] Veazey R Pilch-Cooper H Hope T Alter G Carias A Wang X. Prevention of SHIV transmission by topical IFN-β treatment. Mucos Immunol. (2016) 9:1528–36. 10.1038/mi.2015.146 26838048 PMC4972705

[242] Foster TL Wilson H Iyer SS Coss K Doores K Smith S. Resistance of transmitted founder HIV-1 to IFITM-mediated restriction. Cell Host Microbe. (2016) 20:429–42. 10.1016/j.chom.2016.08.006 27640936 PMC5075283

[243] Iyer SS Bibollet-Ruche F Sherrill-Mix S Learn GH Plenderleith L Smith AG. Resistance to type 1 interferons is a major determinant of HIV-1 transmission fitness. Proc Natl Acad Sci USA. (2017) 114:E590–9. 10.1073/pnas.1620144114 28069935 PMC5278458

[244] Boasso A Hardy AW Anderson SA Dolan MJ Earer G. HIV-induced type I interferon and tryptophan catabolism drive T cell dysfunction despite phenotypic activation. PLoS ONE. (2008) 3:e2961. 10.1371/journal.pone.0002961 18698365 PMC2491901

[245] Yin Z Dai J Deng J Sheikh F Natalia M Shih T. Type III IFNs are produced by and stimulate human plasmacytoid dendritic cells. J Immunol. (2012) 189:2735–45. 10.4049/jimmunol.1102038 22891284 PMC3579503

[246] Tian R-RR Guo H-XX Wei J-FF Yang C-KK He S-HH Wang J-HH. IFN-λ inhibits HIV-1 integration and post-transcriptional events *in vitro*, but there is only limited *in vivo* repression of viral production. Antiviral Res. (2012) 95:57–65. 10.1016/j.antiviral.2012.04.011 22584351

[247] Fonteneau J-FF Larsson M Beignon A-SS McKenna K Dasilva I Amara A. Human immunodeficiency virus type 1 activates plasmacytoid dendritic cells and concomitantly induces the bystander maturation of myeloid dendritic cells. J Virol. (2004) 78:5223–32. 10.1128/JVI.78.10.5223-5232.2004 15113904 PMC400371

[248] Simmons DP Wearsch PA Canaday DH Meyerson HJ Liu YC Wang Y. Type I IFN drives a distinctive dendritic cell maturation phenotype that allows continued class II MHC synthesis and antigen processing. J Immunol. (2012) 188:3116–26. 10.4049/jimmunol.1101313 22371391 PMC3311734

[249] Nabel G Baltimore D. An inducible transcription factor activates expression of human immunodeficiency virus in T cells. Nature. (1987) 326:711–3. 10.1038/326711a0 3031512

[250] Shang L Smith A Reilly C Duan L Perkey K Wietgrefe S. Vaccine-modified NF-kB and GR signaling in cervicovaginal epithelium correlates with protection. Mucos Immunol. (2018) 11:512–22. 10.1038/mi.2017.69 28792003 PMC5807226

[251] Azzoni L Foulkes AS Papasavvas E Mexas AM Lynn KM Mounzer K. Pegylated Interferon alfa-2a monotherapy results in suppression of HIV type 1 replication and decreased cell-associated HIV DNA integration. J Infect Dis. (2013) 207:213–22. 10.1093/infdis/jis663 23105144 PMC3532820

[252] Olesen R Vigano S Rasmussen TA Søgaard OS Ouyang Z Buzon M. Innate immune activity correlates with CD4 T cell-associated HIV-1 DNA decline during latency-reversing treatment with panobinostat. J Virol. (2015) 89:10176–89. 10.1128/JVI.01484-15 26223643 PMC4580197

[253] Li P Kaiser P Lampiris HW Kim P Yukl SA Havlir DV. Stimulating the RIG-I pathway to kill cells in the latent HIV reservoir following viral reactivation. Nat Med. (2016) 22:807–11. 10.1038/nm.4124 27294875 PMC5004598

[254] Sun H Buzon MJ Shaw A Berg RK Yu XG Ferrando-Martinez S. Hepatitis C therapy with interferon-α and ribavirin reduces CD4 T-cell-associated HIV-1 DNA in HIV-1/hepatitis C virus-coinfected patients. J Infect Dis. (2014) 209:1315–20. 10.1093/infdis/jit628 24277743 PMC3982848

[255] Morón-López S Gómez-Mora E Salgado M Ouchi D Puertas MC Urrea V. Short-term treatment with interferon alfa diminishes expression of HIV-1 and reduces CD4+ T-cell activation in patients coinfected with HIV and hepatitis C virus and receiving antiretroviral therapy. J Infect Dis. (2016) 213:1008–12. 10.1093/infdis/jiv521 26525407 PMC4760418

[256] Liu S-YY Sanchez DJ Aliyari R Lu S Cheng G. Systematic identification of type I and type II interferon-induced antiviral factors. Proc Natl Acad Sci USA. (2012) 109:4239–44. 10.1073/pnas.1114981109 22371602 PMC3306696

[257] Kumar NA van der Sluis Mota T Pascoe R Evans VA Lewin SR. Myeloid dendritic cells induce HIV latency in proliferating CD4+ T cells. J Immunol. (2018) 201:1468–77. 10.4049/jimmunol.1701233 30030324 PMC6103814

[258] Evans VA Kumar N Filali A Procopio FA Yegorov O Goulet J-PP. Myeloid dendritic cells induce HIV-1 latency in non-proliferating CD4+ T cells. PLoS Pathogens. (2013) 9:e1003799. 10.1371/journal.ppat.1003799 24339779 PMC3855553

[259] Sedaghat AR German J Teslovich TM Cofrancesco J Jie CC Talbot C. Chronic CD4+ T-cell activation and depletion in human immunodeficiency virus type 1 infection: type I interferon-mediated disruption of T-cell dynamics. J Virol. (2008) 82:1870–83. 10.1128/JVI.02228-07 18077723 PMC2258719

[260] Rotger M Dang KK Fellay J Heinzen EL Feng S Descombes P. Genome-wide mRNA expression correlates of viral control in CD4+ T-cells from HIV-1-infected individuals. PLoS Pathogens. (2010) 6:e1000781. 10.1371/journal.ppat.1000781 20195503 PMC2829051

[261] Hardy GA Sieg S Rodriguez B Anthony D Asaad R Jiang W. Interferon-α is the primary plasma type-I IFN in HIV-1 infection and correlates with immune activation and disease markers. PLoS ONE. (2013) 8:e56527. 10.1371/journal.pone.0056527 23437155 PMC3577907

[262] Dutrieux J Fabre-Mersseman V Muylder B Rancez M Ponte R Rozlan S. Modified interferon-α subtypes production and chemokine networks in the thymus during acute simian immunodeficiency virus infection, impact on thymopoiesis. AIDS. (2014) 28:1101–13. 10.1097/QAD.0000000000000249 24614087

[263] Fitzgerald-Bocarsly P Jacobs ES. Plasmacytoid dendritic cells in HIV infection: striking a delicate balance. J Leukocyte Biol. (2010) 87:609–20. 10.1189/jlb.0909635 20145197 PMC2858309

[264] Li G Cheng M Nunoya J-I Cheng L Guo H Yu H. Plasmacytoid dendritic cells suppress HIV-1 replication but contribute to HIV-1 induced immunopathogenesis in humanized mice. PLoS Pathogens. (2014) 10:e1004291. 10.1371/journal.ppat.1004291 25077616 PMC4117636

[265] O'Brien M Manches O Sabado RL Baranda SJ Wang Y Marie I Rolnitzky L Markowitz M Margolis DM Levy D. Spatiotemporal trafficking of HIV in human plasmacytoid dendritic cells defines a persistently IFN-α-producing and partially matured phenotype. The Journal of clinical investigation. (2011) 121:1088–101. 10.1172/JCI44960 21339641 PMC3049388

[266] O'Brien M Manches O Wilen C Gopal R Huq R Wu V. CD4 receptor is a key determinant of divergent HIV-1 sensing by plasmacytoid dendritic cells. PLoS Pathogens. (2016) 12:e1005553. 10.1371/journal.ppat.1005553 27082754 PMC4833349

[267] Loré K Smed-Sörensen A Vasudevan J Mascola JR Koup RA. Myeloid and plasmacytoid dendritic cells transfer HIV-1 preferentially to antigen-specific CD4+ T cells. J Exp Med. (2005) 201:2023–33. 10.1084/jem.20042413 15967828 PMC2212038

[268] Sandgren KJ Smed-Sörensen A Forsell MN Soldemo M Adams WC Liang F. Human plasmacytoid dendritic cells efficiently capture HIV-1 envelope glycoproteins via CD4 for antigen presentation. J Immunol. (2013) 191:60–9. 10.4049/jimmunol.1202489 23729440 PMC4471340

[269] Hoeffel G Ripoche A-CC Matheoud D Nascimbeni M Escriou N Lebon P. Antigen crosspresentation by human plasmacytoid dendritic cells. Immunity. (2007) 27:481–92. 10.1016/j.immuni.2007.07.021 17869134

[270] Yonezawa A Morita R Takaori-Kondo A Kadowaki N Kitawaki T Hori T. Natural alpha interferon-producing cells respond to human immunodeficiency virus type 1 with alpha interferon production and maturation into dendritic cells. J Virol. (2003) 77:3777–84. 10.1128/JVI.77.6.3777-3784.2003 12610152 PMC149544

